# Combined models for pre- and post-treatment longitudinal biomarker data: an application to CD4 counts in HIV-patients

**DOI:** 10.1186/s12874-016-0187-2

**Published:** 2016-09-15

**Authors:** Oliver T. Stirrup, Abdel G. Babiker, Andrew J. Copas

**Affiliations:** MRC Clinical Trials Unit at UCL, Institute of Clinical Trials & Methodology, University College London, 125 Kingsway, London, WC2B 6NH UK

**Keywords:** CD4, HAART, HIV, Longitudinal data, Mixed effects models, Statistical methodology

## Abstract

**Background:**

There has been some debate in the literature as to whether baseline values of a measurement of interest at treatment initiation should be treated as an outcome variable as part of a model for longitudinal change or instead used as a predictive variable with respect to the response to treatment. We develop a new approach that involves a combined statistical model for all pre- and post-treatment observations of the biomarker of interest, in which the characteristics of response to treatment are treated as a function of the ‘true’ value of the biomarker at treatment initiation.

**Methods:**

The modelling strategy developed is applied to a dataset of CD4 counts from patients in the UK Register of HIV Seroconverters (UKR) cohort who initiated highly active antiretroviral therapy (HAART). The post-HAART recovery in CD4 counts for each individual is modelled as following an asymptotic curve in which the speed of response to treatment and long-term maximum are functions of the ‘true’ underlying CD4 count at initiation of HAART and the time elapsed since seroconversion. Following previous research in this field, the models developed incorporate non-stationary stochastic process components, and the possibility of between-patient differences in variability over time was also considered.

**Results:**

A variety of novel models were successfully fitted to the UKR dataset. These provide reinforcing evidence for findings that have previously been reported in the literature, in particular that there is a strong positive relationship between CD4 count at initiation of HAART and the long-term maximum in each patient, but also reveal potentially important features of the data that would not have been easily identified by other methods of analysis.

**Conclusion:**

Our proposed methodology provides a unified framework for the analysis of pre- and post-treatment longitudinal biomarker data that will be useful for epidemiological investigations and simulations in this context. The approach developed allows use of all relevant data from observational cohorts in which many patients are missing pre-treatment measurements and in which the timing and number of observations vary widely between patients.

**Electronic supplementary material:**

The online version of this article (doi:10.1186/s12874-016-0187-2) contains supplementary material, which is available to authorized users.

## Background

In medical research, there is often interest in evaluating response to treatment conditional on the baseline value at initiation of the biomarker under investigation. In the setting of randomised controlled trials (RCTs), designed primarily to assess the difference between treatment conditions, some authors have argued that optimal efficiency is gained by treating the baseline measurement as an outcome variable within a parametric model [1, 2], whilst Senn has argued that conditioning estimation of treatment effect on the baseline observation through the use of ANCOVA is preferable in most trial situations [3] and Kenward et al. demonstrated that with correct adjustments for sample size the two approaches have nearly identical properties [4]. However, both of these approaches can be problematic when applied to the estimation of response to treatment using longitudinal observational datasets, in which the timing and choice of treatment have not been randomised and in which baseline observations immediately prior to treatment may not be available for all patients. Furthermore, there is often substantial interest in the influence of the baseline value of the biomarker in itself in determining the level of response to treatment, rather than just using this to provide a better estimate of the differences between treatment choices. In this article we describe the development of flexible parametric models for this situation, providing a combined analysis of pre- and post-treatment data in which the response of the biomarker to treatment is dependent on a ‘true’ baseline value that is not directly observed; this combines elements of both previous approaches in that the pre-treatment data are modelled as ‘response variables’, but the trajectory of the biomarker after treatment initiation can also be modelled using flexible functions of the baseline value. The models developed are applied to CD4 cell counts in human immunodeficiency virus (HIV)-positive patients who initiate highly active antiretroviral therapy (HAART).

CD4 cells are a type of white blood cell for which counts are monitored over time both before and after treatment initiation in HIV patients in order to evaluate the progress of the disease and state of the immune system. Although the CD4 counts within an individual can vary erratically over time, on average the counts decline steadily from normal levels following HIV infection and then in most cases recover towards normal levels following initiation of HAART. Over the last 20 years, effective regimens of HAART have been developed for the treatment of HIV, allowing long-term management of the condition and greatly improving the life expectancy and quality of life of affected individuals, at least for those with the condition diagnosed in a resource-rich country. Until recently, clinical guidelines regarding the initiation of treatment varied between countries. In the USA, the Health and Human Services Panel on Antiretroviral Guidelines for Adults and Adolescents have for a number of years recommended immediate initiation of HAART for most patients newly diagnosed with HIV [5], whereas in Europe guidelines recommended monitoring of CD4 in most patients, with treatment initiated once this dropped below 350 [6]. However, a recent RCT has provided definitive evidence of the benefit of immediate initiation of HAART on diagnosis of HIV [7], leading to a shift in clinical guidelines towards early treatment initiation in all well-resourced countries, including the UK [8].

In observational datasets, the timing of recorded CD4 measurements can be highly variable between patients. In much of the existing literature about the long-term response of CD4 counts to HAART, the investigators have avoided any associated complications in their analyses by converting the available data into a set of discrete time points, typically corresponding to annual or 6-monthly observations. This has been done by linear interpolation (Kaufmann et al.) [9], selecting only the observation closest to the chosen time point (Moore and Keruly) [10] or taking the mean measurement within intervals (Lok et al.) [11]. Each of these studies included an analysis stratified by intervals of baseline CD4 count and, although the statistical methodology varied between studies, each found that higher baseline CD4 counts were associated with higher values after several years of HAART. A study by Le et al. suggested that the long-term response to HAART in HIV-positive patients is improved if it is initiated within the first few months after infection, with this effect independent of the CD4 count at baseline [12]. This analysis also relied on stratification of patients into groups.

We now also know that early treatment of HIV leads to a substantial reduction in the occurrence of both acquired immune deficiency syndrome (AIDS)-defining conditions and serious non-AIDS events [7], but there nonetheless remains clinical interest in understanding the factors that are predictive of the recovery in CD4 counts upon HAART initiation as for many patients there is a substantial delay between infection and diagnosis and suboptimal CD4 recovery remains a concern for patients and clinicians [13]. The principal aim of this research is the development of a flexible parametric framework for the combined modelling of pre- and post-treatment CD4 data in HIV positive individuals. This is motivated by the clinical interest in investigating the factors that determine the characteristics of long-term response to HAART, in particular the influences of baseline CD4 count and the time elapsed from infection to treatment initiation. However, the modelling strategy developed could also be used in other settings in which a biomarker is monitored prior to some treatment initiation or clinical intervention.

The modelling strategy described in this article represents a flexible extension of established non-linear mixed effects models, fitted through maximum likelihood estimation based on all observed data using time as a continuous variable. As well as allowing inclusion of all available data in its original format (other than global transformations for normalisation) and the combined assessment of multiple predictive factors, the approach will have the advantage that the characteristics of CD4 trajectories of individual patients over time will be quantified, creating a complete framework for epidemiological simulations or patient-specific predictions, whereas previously this has been done using separate models for pre- and post-treatment data [14]. The models developed are applied to CD4 data from the UK Register of HIV Seroconverters cohort [15]. Following previous work on the modelling of pre-treatment CD4 counts [16], we also incorporate stochastic process components and between-patient differences in variability over time into the models developed. This is done with the aim of defining models that are as realistic as possible in representing the structure of the biological measurements under investigation, which is particularly important when considering analyses for datasets in which missing data and irregular follow-up times are a substantial concern.

## Methods

### Dataset

The UK Register of HIV Seroconverters is an observational cohort study of patients whose date of infection can be reliably estimated [15]. The UK Register of HIV Seroconverters has research ethics approval (MRC MREC: 04/Q2707/155). Recruitment to the cohort began in 1994, but, as we are interested in modelling the response to modern HAART regimens, we restrict our analysis to patients with an estimated date of HIV-1 seroconversion during or after 2003. Patients who started a suboptimal regimen of antiretroviral drugs prior to HAART were excluded, as were patients without at least one post-treatment CD4 count recorded. Patients without any pre-treatment CD4 counts were, however, included in the analysis. HAART is defined by a regimen of at least three antiretroviral drugs from at least two different classes (unless abacavir or tenofovir is used in a regimen with three nucleoside analog reverse-transcriptase inhibitors (NRTIs)).

Application of these conditions resulted in a study population of 852 patients, with a total of 5805 pre-HAART and 7302 post-HAART CD4 observations recorded. The median (interquartile range (IQR)) number of pre-HAART CD4 counts was 5 (3–10), whilst that for post-HAART observations was 6 (3–12). There were a total of 39 patients without any pre-HAART CD4 counts recorded. The median (IQR) time from estimated date of seroconversion to initiation of HAART was 1.3 (0.6–2.8) years, with 192 patients starting HAART within 6 months and 149 starting between 6 months and 1 year from seroconversion.

**Fig. 1:**
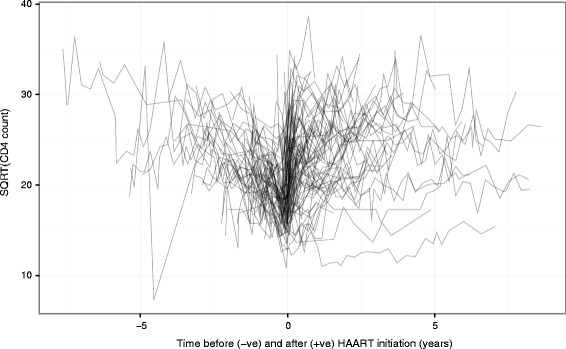
‘Spaghetti plot’ of the square root of CD4 counts from a random sample of 100 patients. Patients are from the UK Register of HIV Seroconverters dataset. Lines are semi-translucent to aid visualisation. Time has been centred at the time of highly active antiretroviral therapy (HAART) initiation for each patient

CD4 cell counts are measured as cells per microlitre, and we followed established practice in modelling the counts on a square-root scale [14, 16]. For the pre-treatment part of the model, time is measured in years from date of HIV seroconversion, whilst for the post-treatment part of the model it is measured in years from HAART initiation. We have censored patients at recorded interruption of HAART (including switch to suboptimal treatment) for more than 1 week, but have not censored according to viral load status or change to HAART regimen. Treatment interruption was recorded in 124 (14.6 %) patients, and there were a total of seven deaths recorded (three of which occurred after censoring due to interruption of HAART). Data from a random subset of 100 of the patients analysed are shown in Fig. 1.

### Baseline state as a latent variable

It can be shown that in situations in which the initiation of treatment is conditional on a biomarker that is monitored over time, and which is measured with error, the observed value of the biomarker at the start of treatment provides a biased estimate of the ‘true’ underlying value [14]. This presents a problem when attempting to model treatment response conditional on the baseline value. We propose that one option in this situation is to build a combined model for both the pre- and post-treatment data, allowing the response to treatment to be conditional on all available pre-treatment data rather than on just a single baseline value. Such an approach would also have the advantage that patients could be included for whom no measurement close to the start of treatment had been obtained. Additionally, fewer assumptions regarding the marginal distribution of ‘true’ baseline values of any given population would be required. For example, such an approach could appropriately deal with a set of distinct treatment initiation guidelines applied across different periods of time or sub-populations, which might lead to a multimodal distribution of baseline values in the total study population, whereas a standard mixed model approach would generally assume the observed baseline values to follow a normal distribution for the population as a whole.

Any linear mixed effects model implies a marginal multivariate normal distribution [17] (*MVN*), for which the log-likelihood function can be expressed in closed form. However, this is not true (except for some special cases) for non-linear mixed effects models [18]. For such models, numerical integration or analytical approximation of the log-likelihood is required at each iteration of any optimisation algorithm [19]. Among the available options, adaptive Gauss–Hermite quadrature is particularly attractive as an increasing number of quadrature points can be used for each random effect to ensure that the log-likelihood is evaluated to an adequate degree of accuracy. However, if more than one random effect is included in the model for each independent individual in the analysis then the number of points that need to be evaluated in the adaptive Gauss–Hermite quadrature algorithm increases exponentially with the number of random effects terms per individual. As such, adaptive Gauss–Hermite quadrature is not generally used when there are more than two or three random effects terms defined in a model, and the computational requirements to attain high accuracy in calculation of the log-likelihood function are lowest when there is only one random effect term per individual.

Because of the computational issues described, to undertake the combined modelling of pre- and post-treatment CD4 data we focus on the use of non-linear latent variable models that require numerical integration only over the unobserved ‘true’ CD4 count at treatment initiation (which we will term *u*). The rationale of this approach is that it will allow adequate flexibility in model structure without increasing the computational requirements to a level that will prevent application to the dataset available. In order to achieve this, we will specify linear mixed models for the pre-treatment data (***y***_*pre*_) and non-linear models for the post-treatment data (***y***_*post*_), conditioned on the ‘true’ baseline CD4 count, that *are* linear in any other random effects terms (allowing a closed form expression for each of these two parts of the model). Under such a scheme, the likelihood function for the combined pre- and post-treatment data for each individual can therefore be expressed as:

$${{\begin{aligned} {}f \left(\boldsymbol{y}_{pre}, \boldsymbol{y}_{post} \right) &= \int_{-\infty}^{\infty}f_{pre,post,u} \left(\boldsymbol{y}_{pre}, \boldsymbol{y}_{post}, u \right) du \\ &= \int_{-\infty}^{\infty}f_{pre} \left(\boldsymbol{y}_{pre} \right) f_{post,u} \left(\boldsymbol{y}_{post}, u | \boldsymbol{y}_{pre} \right) du \\ &= \int_{-\infty}^{\infty}f_{pre} \left(\boldsymbol{y}_{pre} \right) f_{post} \left(\boldsymbol{y}_{post} | {\boldsymbol{y}}_{pre}, u \right) f_{u} \left(u | \boldsymbol{y}_{pre} \right) du. \end{aligned}}} $$

For simplicity above, we suppress notation to indicate that each element of the likelihood function is dependent on model parameters. However, we now consider calculation of the likelihood function dependent on the values of a parameter vector relating to the pre-treatment part of the model ‘ ***θ***_*pre*_’, a parameter vector relating to the post-treatment part of the model ‘ ***θ***_*post*_’ and a shared measurement error variance parameter ‘ *σ*^2^’. If we assume that the post-treatment response depends on the pre-treatment data only though the true baseline value at treatment initiation, i.e. that ***y***_*post*_ is independent of ***y***_*pre*_ given *u*, then we may write:

$${\begin{aligned} {}f \left(\boldsymbol{y}_{pre}, \boldsymbol{y}_{post} \right) &= \int_{-\infty}^{\infty}f_{pre} \left(\boldsymbol{y}_{pre}| \boldsymbol{\theta}_{pre}, \sigma^{2} \right)\\ &\quad f_{post} \left(\boldsymbol{y}_{post} | u, \boldsymbol{\theta}_{post}, \sigma^{2} \right) f_{u} \left(u | {\boldsymbol{y}}_{pre}, \boldsymbol{\theta}_{pre}, \sigma^{2} \right) du. \end{aligned}} $$

This follows a similar form to the likelihood expression for standard random effects models but here the distribution of the latent variable *u*, which is integrated out to obtain the marginal likelihood, is conditioned on the pre-treatment data for each individual rather than following a pre-specified distribution across the population. For those patients in whom no pre-treatment observations were obtained, the likelihood contribution can be calculated solely for the post-treatment observations:

$$\begin{array}{*{20}l} {} f \left(\boldsymbol{y}_{post} \right) &= \int_{-\infty}^{\infty} f_{post} \left(\boldsymbol{y}_{post} | u, \boldsymbol{\theta}_{post}, \sigma^{2} \right) f_{u} \left(u | \boldsymbol{\theta}_{pre}, \sigma^{2} \right) du. \end{array} $$

It should be pointed out here that, in practice, optimisation algorithms to obtain maximum likelihood estimates operate on the log-likelihood scale. In Subsection “Differences in variability between patients”, we describe the addition of two further latent variables to the model for each individual in order to allow for between-patient differences in variability over time.

### Pre-treatment model structure

At present we consider only linear mixed model formulations for the likelihood of ***y***_*pre*:*i*_, representing the observed vector of *n*_*pre*:*i*_ pre-treatment observations for the *i*^th^ individual. However, this is inclusive of stochastic Gaussian process components, such as Brownian motion [20, 21] or fractional Brownian motion [16], as these do not prevent the use of a (multivariate normal) closed form for the pretreatment likelihood function *f*_*pre*_. Denoting the vector of values of the stochastic process **W**_*pre*:*i*_ at times **t**_*pre*:*i*_, and defining ***Σ***_*pre*:*i*_ as the covariance matrix resulting from the chosen Gaussian process for the *i*^th^ individual, the linear mixed model can then be expressed as:

$$\begin{array}{*{20}l} \mathbf{y}_{pre:i} &= \mathbf{X}_{i} \boldsymbol{\beta} + \mathbf{Z}_{i} \mathbf{b}_{i} + \mathbf{W}_{pre:i} + \mathbf{e}_{pre:i} \\ \mathbf{b}_{i} &\sim MVN(\boldsymbol{0}, \, \boldsymbol{\Psi}) \\ \mathbf{W}_{pre:i} &\sim MVN(\boldsymbol{0}, \, \boldsymbol{\Sigma}_{pre:i}) \\ \mathbf{e}_{pre:i} &\sim MVN(\boldsymbol{0}, \, \sigma^{2}\mathbf{I}_{n_{pre:i}}).\end{array} $$

Here, **X**_*i*_ represents the pre-treatment design matrix for the ‘fixed effects’ parameters ***β***, **Z**_*i*_ represents the subset of the columns of the design matrix associated with the pre-treatment ‘random effects’ for each individual **b**_*i*_ and **e**_*pre*:*i*_ is the vector of residual errors for each pre-treatment measurement occasion. The vectors of random effects **b**_1_,**b**_2_⋯**b**_*N*_, residual errors **e**_*pre*:1_,**e**_*pre*:2_⋯**e**_*pre*:*N*_ and stochastic process realisations **W**_*pre*:1_,**W**_*pre*:2_⋯**W**_*pre*:*N*_ for each of the *N* individuals are independent of one another. It can be easily shown that this formulation leads to the following marginal distribution for **y**_*pre*:*i*_:

$$\begin{array}{*{20}l} \mathbf{y}_{pre:i} &\sim MVN\left(\mathbf{X}_{i} \boldsymbol{\beta},\; \mathbf{Z}_{i} \boldsymbol{\Psi} \mathbf{Z}_{i}^{\mathrm{T}} \, + \, \boldsymbol{\Sigma}_{pre:i} \, + \, \sigma^{2}\mathbf{I}_{n_{pre:i}}\right). \end{array} $$

We shall use **V**_*pre*:*i*_ to denote the marginal covariance matrix for **y**_*pre*:*i*_.

In this analysis, we shall consider only a ‘random intercepts and slopes’ structure for the fixed and random effects parts of the pre-treatment model. We shall also include fractional Brownian motion as a Gaussian process component, along with an independent residual error term [16]. A Brownian motion process represents an unpredictable ‘random walk’, and it has been found that adding this as a further component to linear mixed models for pre-treatment CD4 counts in HIV patients leads to an improvement in model fit [20, 21]. Fractional Brownian motion is a generalisation of the standard Brownian motion process [22]. The characteristics of a fractional Brownian motion process are determined by an additional parameter, termed *H* or ‘the Hurst index’, that can take a value in the range (0,1). Standard Brownian motion represents a special case of fractional Brownian motion, corresponding to $H=\frac {1}{2}$. When $H<\frac {1}{2}$, successive increments of the process are negatively correlated. This leads to the path of the trajectory appearing ‘jagged’ and realisations of the process tend to revert towards the mean of zero.

As for standard Brownian motion, the expectation of a fractional Brownian motion process is zero for all points in time (0, *s*, *t* …). A positive scale parameter (*κ*) can be added to the standard definition of fractional Brownian motion, corresponding to the variance of the process at *t*=1. Fractional Brownian motion is a Gaussian process, with the following properties (which determine the structure of ***Σ***_*pre*:*i*_ and ***Σ***_*post*:*i*_):

$$\begin{array}{*{20}l} W_{0}&=0 \\ \mathrm{E}[W_{t}]&=0 \\ \text{Var}[W_{t}]&=\kappa \left|t \right|^{2H} \\ \text{Cov}[W_{s},W_{t}]&= \frac{\kappa}{2} \left(\left|s \right|^{2H} + \left|t \right|^{2H} - \left|t-s \right|^{2H} \right). \end{array} $$

### Conditional distribution of ‘true’ baseline

The use of a pre-treatment model with marginal multivariate normal distribution means that the conditional distribution of the ‘true’ baseline value (*u*_*i*_) at treatment initiation for each individual given their observed pre-treatment data can be readily obtained. We denote the time of treatment initiation from the start of observation (HIV seroconversion in this case) as *t*_*trt*:*i*_. We shall assume that *u*_*i*_ is formed by the sum of the fixed effects parameter vector (***β***) multiplied by a row vector (**X**_*trt*:*i*_) corresponding to an extension of the design matrix (**X**_*i*_) for that individual relating to variable values (e.g. time) at *t*_*trt*:*i*_, the equivalent term for the subject-specific random effects (i.e. **Z**_*trt*:*i*_**b**_*i*_) and the realisation of the subject’s stochastic process at *t*_*trt*:*i*_:

$$\begin{array}{*{20}l} u_{i} &= \mathbf{X}_{trt:i} \boldsymbol{\beta} + \mathbf{Z}_{trt:i} \mathbf{b}_{i} + W_{trt:i}. \end{array} $$

As such, the joint distribution **y**_*pre*:*i*_ and *u*_*i*_ is multivariate normal:

$${{ \begin{aligned} &{}\left(\begin{array}{cc} \mathbf{y}_{pre:i} \\ u_{i} \end{array} \right)\sim MVN \left(\left(\begin{array}{cc} \mathbf{X}_{i} \boldsymbol{\beta} \\ \mathbf{X}_{trt:i} \boldsymbol{\beta} \end{array}\right),\right.\\ &{} \left.\left(\begin{array}{cc} \mathbf{V}_{pre:i} & \!\mathbf{Z}_{i} \boldsymbol{\Psi} \mathbf{Z}_{trt:i}^{\mathrm{T}} \,+\, \text{Cov} \left[ \mathbf{W}_{pre:i},\! W_{trt:i} \right] \\ \! \mathbf{Z}_{trt:i} \boldsymbol{\Psi} \mathbf{Z}_{i}^{\mathrm{T}} \,+\, \text{Cov} \left[ W_{trt:i}, \mathbf{W}_{pre:i} \right] &\! \mathbf{Z}_{trt:i} \boldsymbol{\Psi} \mathbf{Z}_{trt:i}^{\mathrm{T}} \,+\, \text{Var} \left[ W_{trt:i} \right] \end{array}\!\!\right)\! \!\right)\!. \end{aligned}}} $$

The variance and covariance terms for the stochastic component of the model can be calculated for any given Gaussian process based on **t**_*pre*:*i*_, *t*_*trt*:*i*_ and any pre-treatment model parameters relating to the process. The conditional probability density function of *u*_*i*_ given **y**_*pre*:*i*_, *f*_*u*_(*u*_*i*_|***y***_*pre*:*i*_,***θ***_*pre*_,*σ*^2^), can therefore be obtained using the standard result for a partitioned multivariate normal distribution. Using a simplified notation:

$$\left(\begin{array}{cc} \mathbf{y}_{pre:i} \\ u_{i} \end{array}\right) \sim MVN \left(\left(\begin{array}{cc} \mathbf{X}_{i} \boldsymbol{\beta} \\ \mathbf{X}_{trt:i} \boldsymbol{\beta} \end{array}\right), \left(\begin{array}{cc} \mathbf{V}_{pre:i} & \mathbf{v}_{12:i}\\ \mathbf{v}_{21:i} & v_{22:i} \end{array} \right) \right), $$

it is known that:

$$\begin{array}{*{20}l} u_{i} | \mathbf{y}_{pre:i} &\sim N \left(\mu\prime, v\prime \right), \\ \text{where} \,\mu\prime &= \mathbf{X}_{trt:i} \boldsymbol{\beta} + \mathbf{v}_{21:i} \mathbf{V}_{pre:i}^{-1} \left(\mathbf{y}_{pre:i} - \mathbf{X}_{i} \boldsymbol{\beta} \right) \\ \text{and}\, v\prime &= v_{22:i} - \mathbf{v}_{21:i} \mathbf{V}_{pre:i}^{-1} \mathbf{v}_{12:i}. \end{array} $$

If a patient has no pre-treatment observations, then the probability density function for the baseline value is simply that for a normal distribution with mean **X**_*trt*:*i*_***β*** and variance *v*_22:*i*_.

The conditional distribution of each *u*_*i*_ is normal and so will include potential negative realisations, even if the probability of this is vanishingly small for most individuals. As such, we use the notation $u_{i}^{+}$ to indicate a latent variable for which all probability mass for values *u*_*i*_<0 is assigned instead to *u*_*i*_=0, i.e. $u_{i}^{+} = Max\left (0, u_{i} \right)$. The coding used to achieve this is given in Additional file 1.

### Post-treatment model structure

#### Mean response to treatment

Although a range of models could be considered for the post-treatment observations, we focus on the use of an asymptotic regression model for the underlying mean structure. Such models have been used to describe CD4 recovery over several years from treatment initiation in children [23, 24]. In our definition of this model, the mean value for the *i*^th^ individual at time after initiation of treatment *t*_*post*_, conditional on the ‘true’ baseline value $u_{i}^{+}$, is given by the function:

1 $$ \begin{aligned}  g \left(t_{post}, u_{i}^{+} \right) &= \phi_{1:i} + \left(u_{i}^{+} - \phi_{1:i} \right) \exp \left(-\exp \left(\phi_{2:i} \right) t_{post} \right). \end{aligned}  $$

**Fig. 2:**
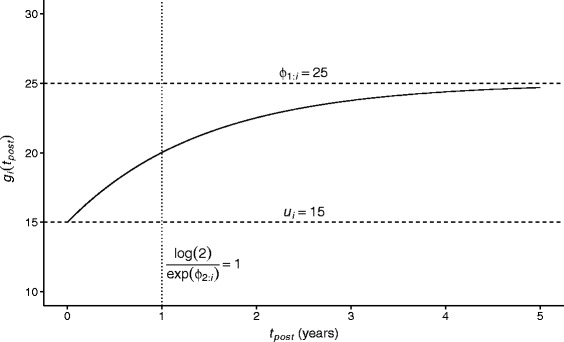
Illustrative plot of an asymptotic regression curve. Here the baseline (*u*_*i*_) is set to 15, the asymptotic maximum (*ϕ*_1:*i*_) is set to 25 and the rate of recovery parameter (*ϕ*_2:*i*_) is set to log(log(2)), leading to a ‘half-life’ of 1

This function takes the value $u_{i}^{+}$ when *t*_*post*_=0 (i.e. at the exact time of treatment initiation), and it has a horizontal asymptote at *ϕ*_1:*i*_ as *t*_*post*_→*∞*. The value of *ϕ*_2:*i*_ determines the speed of transition from $u_{i}^{+}$ to *ϕ*_1:*i*_, i.e. from the value of the response variable at baseline to its long-term mean, as *t*_*post*_ increases. The shape of the function is illustrated in Fig. 2. It is useful to note that, as this function involves a change from a baseline value to a long-term maximum that follows an ‘exponential decay’-type curve, the ‘half life’ of this transition can be calculated as $\frac {\log \left (2 \right)}{\exp \left (\phi _{2:i}\right)}$; this facilitates interpretation of the estimated values of parameters that define *ϕ*_2:*i*_.

In models of this type, the place of $u_{i}^{+}$ in this function is usually taken by a single parameter (or a linear function of a set of parameters) to be estimated, potentially with an associated subject-specific random effect term. However, we instead make use of the fact that a subject-specific distribution for $u_{i}^{+}$ can be included in the model conditioned on the observed pre-treatment data for that individual. Similarly, we will consider *ϕ*_1:*i*_ and *ϕ*_2:*i*_ as potentially being determined as a function of $u_{i}^{+}$, alongside other variables, i.e. we will investigate whether the long-term average value of the response variable and the speed at which this is attained are predicted by the ‘true’ value of the variable at treatment initiation.

#### Long-term maximum

The simplest potential model for the long-term maximum response to treatment in each individual, i.e. the horizontal asymptote *ϕ*_1:*i*_, is to assume that this is equal to a single constant for the entire population:

$$\begin{array}{*{20}l} \phi_{1:i} = A_{1} \text{, for all} {i}. \end{array} $$

The implication of this model is that the long-term response to treatment does not depend on the value of the variable in any given patient at treatment initiation, or on any other factors. This formulation also assumes that there is no random variation in the long-term maximum response between patients, but we will include a subject-specific random-effect term ‘ *τ*_*i*_’, alongside any deterministic function (*ϕ*_1_(…)), throughout:

$$\begin{array}{*{20}l} \phi_{1:i} = \phi_{1}\left(\ldots \right) + \tau_{i}, \text{where} \,\tau_{i} \sim N \left(0, P \right), \end{array} $$

with the variance parameter *P* to be estimated. Although the post-treatment model defined in Eq. (1) is non-linear in terms of the parameters, using this formulation it *is* linear in terms of the subject-specific random effect. As such *f*_*post*_(***y***_*post*_|*u*,***θ***_*post*_,*σ*^2^) can be expressed in closed form as a multivariate normal distribution (assuming no further random effect terms are added to the model), even though it does not constitute a linear mixed effects model conditioned on the unobserved baseline variable. Further details are given in Additional file 1.

The next model considered is that the expected long-term maximum (working on the square-root scale for CD4 counts) for any given patient follows a linear dependence on their ‘true’ value at treatment initiation:

$$\begin{array}{*{20}l} \phi_{1}\left(u_{i}^{+} \right) = A_{1} + A_{2} \times u_{i}^{+}. \end{array} $$

Where *A*_1_ and *A*_2_ are parameters to be estimated.

We then wish to investigate whether *ϕ*_1_ is a more complex, non-linear, function of $u_{i}^{+}$. One option would be to specify that *ϕ*_1_ is some specific non-linear function of $u_{i}^{+}$. However, the fact that the relationship between *ϕ*_1:*i*_ and $u_{i}^{+}$ cannot be directly visualised using the raw data means that there is no obvious way to go about selecting the functional form. Another option is the use of cubic splines defined in terms of $u_{i}^{+}$, this approach has the advantage of allowing consideration of a wide variety of possible relationships between the predictive and outcome variable. In order to restrict the total number of model parameters and improve stability of optimisation, we make use of natural cubic splines derived from a truncated power series basis as described by Hastie, Tibshirani and Friedman [25]. We use knots at 15.5, 17.5, 19.5 and 22 in terms of square-root CD4, corresponding to approximately the 20^th^, 40^th^, 60^th^ and 80^th^ centiles of the last observed CD4 count before treatment initiation, when available, in the UK Register of HIV Seroconverters dataset.

We also consider models in which the relationship between the long-term maximum response and the baseline value ($u_{i}^{+}$) can vary according to the time elapsed between seroconversion and treatment initiation for each patient (*t*_*trt*:*i*_). Although ideally this would be done using a smooth function of $u_{i}^{+}$ and *t*_*trt*:*i*_, for computational stability we fit separate functions of $u_{i}^{+}$ stratified by *t*_*trt*:*i*_ (in years) as follows: 0≤*t*_*trt*:*i*_≤0.5, 0.5<*t*_*trt*:*i*_≤1.0 and 1.0<*t*_*trt*:*i*_. These grouping were chosen based on a combination of findings reported previously in the literature, the level of uncertainty in terms of estimated dates of seroconversion in our study population and the need to ensure that an adequate number of patients were included in each group to allow parameter estimates to be obtained for the model.

Were patient characteristics (i.e. age, gender *etc.*) to be included in the model for *ϕ*_1:*i*_, and assuming a linear function in terms of $u_{i}^{+}$ for simplicity of exposition, we would have an extended function for *ϕ*_1_ of the form:

$$\begin{array}{*{20}l} \phi_{1}\left(u_{i}^{+}, \mathbf{x}_{i} \right) = A_{1} + A_{2} \times u_{i}^{+} + \mathbf{x}_{i}^{\mathrm{T}} \boldsymbol{\beta}_{\phi_{1}}, \end{array} $$

where **x**_*i*_ is the patient-specific vector of data specifying relevant characteristics and $\boldsymbol {\beta }_{\phi _{1}}$ is the associated vector of parameters that determines their effects.

#### Speed of response to treatment

As for the function for the long-term maximum value, we consider first a constant value for *ϕ*_2:*i*_ across the population (*ϕ*_2:*i*_=*B*_1_) and secondly a linear dependence on $u_{i}^{+}$:

$$\begin{array}{*{20}l} \phi_{2:i} = B_{1} + B_{2} \times u_{i}^{+} \end{array} $$

where *B*_1_ and *B*_2_ are parameters to be estimated. We then consider a natural cubic spline function of $u_{i}^{+}$, including an analysis with stratification according to groups defined by the time elapsed from seroconversion to treatment. The addition of a subject-specific random effect to this function was also considered, this required integration of the log-likelihood function over an additional latent variable for each patient and so the Laplace approximation was used.

#### Residual variance structure

We propose the following model for the vector of post-treatment observations (**y**_*post*:*i*_) for the *i*^th^ individual, conditioned on their ‘true’ baseline value at treatment initiation ($u_{i}^{+}$):

$$\begin{array}{*{20}l} \mathbf{y}_{post:i}|_{U_{i}^{+}=u_{i}^{+}} &= \mathbf{g} \left(\mathbf{t}_{post:i}, u_{i}^{+}, \tau_{i}\right) + \mathbf{W}_{post:i} + \mathbf{e}_{post:i} \\ \tau_{i} &\sim N\left(0, \, P \right) \\ \mathbf{W}_{post:i} &\sim MVN\left(\boldsymbol{0}, \, \boldsymbol{\Sigma}_{post:i}\right) \\ \mathbf{e}_{post:i} &\sim MVN\left(\boldsymbol{0}, \, \sigma^{2}\mathbf{I}_{n_{post:i}}\right). \end{array} $$

The vector of observation times **t**_*post*:*i*_ relates to time since treatment initiation, with *n*_*post*:*i*_ post-treatment observations for the *i*^th^ subject. The function **g** here represents a vectorised version of *g* in Eq. (1), i.e.:

$$\begin{array}{*{20}l} \mathbf{g} \left(\mathbf{t}_{post:i}, u_{i}^{+}, \tau_{i}\right) = \left(\begin{array}{cc} g \left(t_{post:i1}, u_{i}^{+}, \tau_{i}\right) \\ g \left(t_{post:i2}, u_{i}^{+}, \tau_{i}\right) \\ \vdots \\ g \left(t_{post:in_{post:i}}, u_{i}^{+}, \tau_{i}\right) \end{array}\right). \end{array} $$

For the stochastic process component **W**_*post*:*i*_, we include a ‘new’ fractional Brownian motion process with value zero at time of treatment initiation and separate parameters to the pre-treatment process. The vector **e**_*post*:*i*_ represents independent residual measurement errors (or very short-term physiological variation), with a variance parameter (*σ*^2^) that is shared with the pre-treatment model.

### Differences in variability between patients

Previous work on pre-treatment CD4 counts in HIV patients has found that the generalisation of the model structure as described in “Pre-treatment model structure” to a multivariate-t distribution leads to a substantial improvement in model fit in terms of the log-likelihood and residual diagnostic plots [16]. However, the application of a marginal multivariate-t distribution is not possible in the current setting, in which a combined model is defined for pre- and post-treatment data. We instead consider models in which the stochastic process components before and after treatment each follow a marginal multivariate-t distribution, with correlated scaling variables.

There are a number of multivariate generalisations of the univariate t-distribution, and a thorough review of this topic is provided by Kotz and Nadarajah [26]. However, we refer to the *multivariate-t distribution* as that with the probability density function:

$${{\begin{aligned} {} f\!\left(\! \mathbf{y}_{i} ; \boldsymbol{\mu}_{i}, \!\mathbf{V\!}_{i}, v \right) \,=\, \frac{\boldsymbol{\Gamma} \left(\left(v + n_{i} \right) /2 \right)} {\boldsymbol{\Gamma}\! \!\left(v\!/\!2 \right) \!v^{n_{i}/\!2} \pi^{\!n_{i}/2} \!\left|\! \mathbf{V}_{i} \right|^{1/2}\! \left(\!1 \,+\, \frac{1}{v}\! \!\left(\! \mathbf{y}_{i} \,-\, \boldsymbol{\mu}_{i} \!\right)^{\mathrm{\!T}} \mathbf{\!V}_{i}^{-1}\! \left(\! \mathbf{y}_{i}\! -\! \boldsymbol{\mu}_{i} \!\right)\! \right)^{\!(\!v+n_{i})/2}} \text{\!,} \end{aligned}}} $$

where *n*_*i*_ represents the length of the random vector **y**_*i*_ ($\in \mathbb {R}^{n_{i}}$), **V**_*i*_ is a *n*_*i*_×*n*_*i*_ positive-definite scale matrix, ***μ***_*i*_ is a *n*_*i*_×1 location vector and *v* is a degrees of freedom parameter. The mean of the distribution is ***μ***_*i*_ if *v*>1 and otherwise undefined, and the variance of the distribution is $\frac {v}{v-2} \mathbf {V}_{i}$ if *v*>2 and otherwise undefined.

If a vector of observations **y**_*i*_ follows a multivariate-t distribution:

$$\begin{array}{*{20}l} \mathbf{y}_{i} \sim t_{n_{i}} \left(\mathbf{X}_{i}\boldsymbol{\beta}, \: \mathbf{V}_{i}, \: v \right), \end{array} $$

then this can alternatively be represented as a hierarchical model in which **y**_*i*_ follows a multivariate normal distribution conditional on a gamma-distributed variable *w*_*i*_ (with parameters given for ‘shape’ and ‘rate’, respectively) [27]:

2 $$\begin{array}{*{20}l} \mathbf{y}_{i} \vert_{W_{i}=w_{i}} &\sim MVN \left(\mathbf{X}_{i}{\boldsymbol{\beta}}, \: {\frac{1}{{w}_{i}}} \mathbf{V}_{i} \right)  \\ W_{i} &\sim \text{gamma} \left(\frac{v}{2}, \: {\frac{v}{2}} \right). \notag \end{array} $$

The desired model structure for a combined analysis of pre- and post-treatment data requires the use of a bivariate gamma distribution, of which a number are available (as reviewed by Balakrishna and Lai [28]). Such models will include three latent variables per patient, and as such a Laplace approximation to the log-likelihood [19, 29, 30] rather than adaptive Gauss–Hermite quadrature will be used. Because of this, Moran’s bivariate gamma distribution [28, 31] makes a natural choice. This distribution is defined by first transforming random variables (A and B) from the standard normal bivariate distribution with correlation *ρ*_*Moran*_ into a copula *C*(*Φ*(*a*),*Φ*(*b*)), where *Φ* is the standard normal cumulative distribution function, and secondly using the inverse cumulative distribution functions of univariate gamma distributions (*W*_1_=*F*^−1^(*Φ*(*A*)), *W*_2_=*G*^−1^(*Φ*(*B*))) to find the joint distribution function of *W*_1_ and *W*_2_ (each of which has a marginal univariate gamma distribution). *F* is here defined as the cumulative distribution function for gamma distribution with ‘shape’ and ‘rate’ parameters both equal to $\frac {v_{1}}{2}$, whilst *G* is that for the gamma distribution with parameters both equal to $\frac {v_{2}}{2}$.

Analogous to our previous work [16], the model for pre-treatment CD4 counts is then defined as:

$$\begin{array}{*{20}l} \mathbf{y}_{pre:i} &= \mathbf{X}_{i} \boldsymbol{\beta} + \mathbf{Z}_{i} \mathbf{b}_{i} + \mathbf{W}_{pre:i} + \mathbf{e}_{pre:i}  \\ \mathbf{b}_{i} &\sim MVN(\boldsymbol{0}, \, \boldsymbol{\Psi}) \\ \mathbf{W}_{pre:i}|_{W_{1:i}=w_{1:i}} &\sim MVN\left(\boldsymbol{0}, \, \frac{1}{w_{1:i}}\boldsymbol{\Sigma}_{pre:i}\right) \\ \mathbf{e}_{pre:i} &\sim MVN\left(\boldsymbol{0}, \, \sigma^{2}\mathbf{I}_{n_{pre:i}}\right), \end{array} $$

whilst, the model for post-treatment data is:

$$\begin{array}{*{20}l} \mathbf{y}_{post:i}|_{U_{i}^{+}=u_{i}^{+}} &= \mathbf{g} \left(\mathbf{t}_{post:i}, u_{i}^{+}, \tau_{i}\right) + \mathbf{W}_{post:i} + \mathbf{e}_{post:i} \\ \tau_{i} &\sim N\left(0, \, P \right) \\ \mathbf{W}_{post:i}|_{W_{2:i}=w_{2:i}} &\sim MVN\left(\boldsymbol{0}, \, \frac{1}{w_{2:i}}\boldsymbol{\Sigma}_{post:i}\right) \\ \mathbf{e}_{post:i} &\sim MVN\left(\boldsymbol{0}, \, \sigma^{2}\mathbf{I}_{n_{post:i}}\right), \end{array} $$

with the scaling factors jointly following Moran’s bivariate gamma distribution:

$$\begin{array}{*{20}l} \left(\begin{array}{cc} W_{1:i} \\ W_{2:i} \end{array}\right) &\sim Moran \left(\rho_{Moran}; \frac{v_{1}}{2}, \frac{v_{1}}{2}; \frac{v_{2}}{2}, \frac{v_{2}}{2} \right). \end{array} $$

This specific bivariate gamma distribution is a natural choice because the marginal log-likelihood function for the model can be found by integrating out the latent variables on the standard normal scale, for which the Laplace approximation is optimally accurate [32], as follows (omitting indexing for each individual and dependence on model parameters):

$${{ \begin{aligned} &{}f\! \left(\boldsymbol{y}_{pre}, \boldsymbol{y}_{post} \!\right) =\\ &\!\!\int_{-\infty}^{\infty} \int_{-\infty}^{\infty} \int_{-\infty}^{\infty} \!f_{pre}\! \left(\! \boldsymbol{y}_{pre}|w_{1}\!\,=\,F^{-1}\!\!\left(\! \Phi(a) \!\right)\! \right) f_{post} \!\!\left(\! \boldsymbol{y}_{post} | u, \!w_{2}\,=\,G^{-1}\!\!\left(\!\Phi(b)\! \right)\! \right) \\ &{\kern6pt}f_{u}\! \left(\!u | \boldsymbol{y}_{pre}, w_{1}\!\,=\,F^{-1}\!\left(\Phi(a) \!\right)\! \right) f_{ab}\left(a,b\right) du\ da\ db, \end{aligned}}} $$

where *f*_*ab*_ is the probability density function for a standard bivariate normal distribution with correlation *ρ*_*Moran*_. The *ρ*_*Moran*_ parameter can be estimated from the data through maximum likelihood estimation as for other model parameters.

### Overall model structure and interpretation

**Fig. 3:**
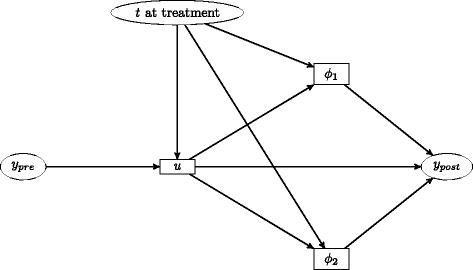
Directed acyclic graph depicting the proposed model structure for each patient. Observed variables are shown within *ellipses*, whilst unobserved latent variables are shown within *rectangles*

A directed acyclic graph depicting the proposed model structure is shown in Fig. 3. For simplicity, we omit here the extension to the basic model in which further latent variables are added to the model to allow between-patient differences in variability over time as described in Subsection “Differences in variability between patients”. This diagram illustrates the fact that in the model, response to treatment is linked to pre-treatment data only through the ‘true’ baseline value *u* and the time from seroconversion to treatment initiation. These links are mediated through variables representing the long-term maximum response to treatment (*ϕ*_1_) and the speed at which this is attained (*ϕ*_2_) in each patient. When fitted to the dataset under investigation, this structure should allow estimates of individual parameters of the model to be interpreted in a meaningful way. Although in this article we do not consider further potential predictive variables, it would be relatively straightforward to extend the model to assess whether patient characteristics such as age and gender or drug regimen choice are independently predictive of response to treatment.

The primary interpretation of our models as presented is the prediction of the response to HAART in terms of prior CD4 counts and time from seroconversion. It has been argued that causal effects can only be estimated from observational studies with respect to clearly defined interventions [33]. Whilst interventions with regard to the monitoring of CD4 counts and guidelines for treatment initiation can be defined within the present context, it is not possible to begin treatment conditional on the ‘true’ value of a patient’s CD4 count, as this cannot be observed directly. Furthermore it is not possible to define a treatment policy in terms of a specific simultaneous combination of ‘time from seroconversion’ and ‘true CD4 count’, when in a certain period a patient may only experience a limited range of CD4 counts.

As we have censored patients at recorded interruption of HAART but not according to viral load status, the fitted models can be taken to represent treatment response for all patients were they all to remain on HAART (regardless of success or failure of virological suppression). All included patients had at least one post-HAART CD4 observation, but beyond this the number and timing of CD4 cell counts recorded for each individual were highly variable. We have assumed that the missingness of observations can be treated as ‘missing at random’ (following the terminology of Rubin [34]), i.e. that the ‘missingness’ of any observation is independent of the unobserved data conditional on the observed values of the outcome variable and any other covariates included in the model. Similarly we assume that the timing of observations is dependent only on previously observed outcomes, under which condition maximum likelihood estimation of a model for the outcome variable alone is consistent, without the need for specification of a model for the distribution of follow-up times [35].

### Maximum likelihood estimation

All models presented have been fitted by direct maximum likelihood estimation using the open source AD Model Builder software (Version 11.2; ADMB Foundation) [30]. This requires the user to write out the log-likelihood function for the model in terms of the data and unknown parameters to be estimated in the C++ language, with additional statistical and mathematical functions (including matrix and vector functions and operations) provided by the software to facilitate this. The ‘random effects’ mode was used for ADMB, allowing optimisation of a log-likelihood function with automated integration over latent variables [29]. The log-likelihood function for each individual (for their complete pre- and post-treatment data) was defined using the ‘separable function’ utility, allowing computational efficiency to be gained from the modelled independence of each individual. 15-point adaptive Gauss–Hermite quadrature was used to obtain the maximum likelihood estimates for all models described in this report for which only one latent variable was included per individual (i.e. the ‘true’ baseline). However, for the models including additional latent variables associated with between-patient differences in variability over time, Gauss–Hermite quadrature was not feasible and the Laplace approximation was used.

Models were parameterised using logarithmic, logistic and generalised logistic transformations where appropriate such that parameter estimates could be obtained using unrestricted optimisation (e.g. maximum likelihood estimation was carried out using log-transformed variance parameters, with a parameter space of (−*∞*,+*∞*) rather than [0,+*∞*]). For all model parameters, confidence intervals are reported derived from the estimated asymptotic multivariate normal sampling distribution based on the observed information on the transformed scales. The ‘R2admb’ package [36] was used to output data files in the necessary format through the R statistical computing environment (R Foundation, Vienna, Austria). The ggplot2 package for R [37] was used for statistical graphics. All maximum likelihood estimates reported in this document were obtained using a computer cluster running with Linux operating systems. The authors acknowledge the use of the University College London (UCL) Legion High Performance Computing Facility (Legion@UCL), and associated support services, in the completion of this work. Fitting each of the models presented to the UK Register of HIV Seroconverters dataset took between 1 and 2$\frac {1}{2}$ hours (using a core with 4GB RAM), whereas fitting one of the models using a mid–low specification personal laptop (4GB RAM, Celeron Dual-Core CPU T3500 @ 2.1 GHz) required around 10 h.

When considering only a single latent variable per patient, nested models are compared using the generalised likelihood ratio test, comparing the change in 2×log-likelihood (*Δ*2*ℓ*) to a *χ*^2^ distribution. Non-nested models are compared using the Bayesian information criterion (BIC) statistic, using the total number of observations in the dataset for the calculation of the penalty term. It is worth noting that these methods are only valid because adaptive Gauss–Hermite quadrature can be used to calculate the log-likelihood of the fitted models to a high degree of accuracy; this is not the case for less computationally intensive approximations of the log-likelihood.

## Results

### Model fitting

Summaries of the set of models fitted to the UK Register of HIV Seroconverters dataset are presented in Table 1, and to facilitate their interpretation Table 2 provides a description of each model parameter. The most basic model considered included constant parameters for the mean long-term maximum CD4 count (on square-root scale) and the rate of recovery from baseline at treatment initiation, without division of patients according to time from seroconversion to initiation of HAART (Model_1_ in Table 1). Modelling the long-term maximum (*ϕ*_1_) and speed of response to treatment (*ϕ*_2_) as linear functions of the baseline value in each individual ($u_{i}^{+}$) led to a significant improvement in model fit (Model_2_*vs* Model_1_, *Δ*2*ℓ* 460.4 for 2 parameters; *P*<0.0001). A model equivalent to Model_2_ but without pre- and post-treatment stochastic process components was also fitted for comparison and was found to have a much higher BIC value (64398); correspondingly the model including stochastic processes showed a significant improvement in fit (*Δ*2*ℓ* 844.8 for 4 parameters; *P*<0.0001). The extension of *Model*_2_ to allow natural cubic spline functions to define the relationships between $u_{i}^{+}$ and *ϕ*_1_ and *ϕ*_2_ led to a further significant improvement in model fit (Model_3_*vs* Model_2_, *Δ*2*ℓ* 31.4 for 4 parameters; *P*<0.0001).

**Table 1:** Summary of the results of combined models for pre- and post- highly active antiretroviral therapy (HAART) CD4 cell count data, after square root transformation, for patients from the UK Register of HIV Seroconverters dataset

	*M* *o* *d* *e* *l*_1_	*M* *o* *d* *e* *l*_2_	*M* *o* *d* *e* *l*_3_	*M* *o* *d* *e* *l*_4_	*M* *o* *d* *e* *l*_5_	*M* *o* *d* *e* *l*_6_
*β*_0_	22.44 (22.13 to 22.74)	22.45 (22.16 to 22.74)	22.44 (22.15 to 22.73)	22.26 (21.96 to 22.56)	22.26 (21.96 to 22.56)	22.23 (21.94 to 22.53)
*β*_1_	−1.36 (−1.52 to −1.2)	−1.39 (−1.55 to −1.23)	−1.39 (−1.55 to −1.23)	−1.3 (−1.46 to −1.14)	−1.32 (−1.47 to −1.16)	−1.36 (−1.5 to −1.21)
*U*_00_	12.37 (10.64 to 14.37)	13.39 (11.77 to 15.23)	13.42 (11.79 to 15.28)	14.43 (12.68 to 16.43)	14.53 (12.77 to 16.54)	12.92 (11.29 to 14.8)
*ρ*	−0.65 (−0.79 to −0.44)	−0.86 (−0.99 to 0.18)	−0.84 (−0.98 to −0.1)	−0.95 (−1 to 1)	−0.92 (−1 to 0.91)	−0.63 (−0.76 to −0.44)
*U*_11_	0.55 (0.33 to 0.93)	0.25 (0.08 to 0.75)	0.28 (0.1 to 0.75)	0.2 (0.05 to 0.74)	0.21 (0.06 to 0.74)	0.49 (0.31 to 0.77)
*κ*_*pre*_	9.68 (8.77 to 10.68)	5.91 (5.23 to 6.67)	5.9 (5.22 to 6.68)	5.99 (5.29 to 6.8)	5.92 (5.21 to 6.72)	5.37 (4.37 to 6.6)
*H*_*pre*_	0.11 (0.09 to 0.14)	0.3 (0.25 to 0.37)	0.3 (0.24 to 0.36)	0.31 (0.25 to 0.37)	0.31 (0.25 to 0.38)	0.16 (0.13 to 0.19)
*σ*	1.25 (1.09 to 1.42)	1.95 (1.89 to 2.01)	1.94 (1.87 to 2)	1.92 (1.85 to 1.99)	1.92 (1.86 to 1.99)	1.32 (1.19 to 1.46)
*ϕ*_1_ model:						
long-term	Constant for all patients	Linear for all patients	NCS for all patients	Linear for all patients	Linear for early treatment	Linear for all patients
maximum				stratified by *A* *R* *T*_*t*_	groups or NCS for late	stratified by *A* *R* *T*_*t*_
					treatment group	
*A* *t*1_1_	—	—	—	7.04 (4.75 to 9.33)	7.06 (4.77 to 9.35)	8.44 (6.05 to 10.83)
*A* *t*1_2_	—	—	—	0.9 (0.79 to 1.01)	0.9 (0.79 to 1)	0.84 (0.72 to 0.95)
*A* *t*2_1_	—	—	—	10.73 (7.93 to 13.53)	10.68 (7.85 to 13.51)	12.32 (9.28 to 15.35)
*A* *t*2_2_	—	—	—	0.67 (0.54 to 0.81)	0.67 (0.53 to 0.81)	0.64 (0.47 to 0.8)
*A*_1_	25.93 (25.49 to 26.36)	11.42 (9.74 to 13.09)	5.1 (0.3 to 9.9)	14.58 (12.3 to 16.86)	3.76 (−1.99 to 9.51)	14.35 (12.32 to 16.38)
*A*_2_	—	0.69 (0.62 to 0.77)	1.14 (0.84 to 1.44)	0.55 (0.44 to 0.66)	1.23 (0.86 to 1.6)	0.57 (0.46 to 0.67)
*A*_3_	—	—	-0.43 (−0.64 to −0.22)	—	−0.32 (−0.63 to −0.01)	—
*A*_4_	—	—	0.82 (0.43 to 1.2)	—	0.52 (−0.07 to 1.11)	—
*ϕ*_2_ model:						
recovery	Constant for all patients	Linear for all patients	NCS for all patients	Linear for all patients	Linear for early treatment	Linear for all patients
speed				stratified by *A* *R* *T*_*t*_	groups or NCS for late	stratified by *A* *R* *T*_*t*_
					treatment group	
*B* *t*1_1_	—	—	—	2.66 (0.52 to 4.79)	2.8 (0.76 to 4.84)	5.68 (2.94 to 8.43)
*B* *t*1_2_	—	—	—	0.02 (−0.08 to 0.11)	0.01 (−0.08 to 0.1)	−0.14 (−0.29 to −1.98e-03)
*B* *t*2_1_	—	—	—	−0.99 (−3 to 1.02)	−0.92 (−2.97 to 1.13)	0.23 (−1.39 to 1.86)
*B* *t*2_2_	—	—	—	0.15 (0.05 to 0.26)	0.15 (0.04 to 0.26)	0.01 (−0.1 to 0.12)
*B*_1_	-0.16 (−0.3 to −0.02)	−3.34 (−4.19 to −2.48)	1.82 (−0.23 to 3.87)	−3.64 (−4.7 to −2.59)	2.42 (0.26 to 4.58)	−2.25 (−3.3 to −1.21)
*B*_2_	—	0.24 (0.2 to 0.28)	−0.11 (−0.24 to 0.02)	0.23 (0.17 to 0.29)	−0.15 (−0.29 to −0.02)	0.13 (0.07 to 0.19)
*B*_3_	—	—	0.28 (0.19 to 0.38)	—	0.19 (0.04 to 0.33)	—
*B*_4_	—	—	−0.52 (−0.71 to −0.33)	—	−0.28 (−0.58 to 0.02)	—
*P*	11.09 (8.76 to 14.03)	2.97 (2.09 to 4.23)	3.05 (2.13 to 4.38)	3.07 (2.19 to 4.31)	3.31 (2.39 to 4.59)	2.72 (1.71 to 4.31)
*κ*_*post*_	7.59 (6.79 to 8.49)	3.09 (2.46 to 3.89)	3.17 (2.53 to 3.98)	3.36 (2.7 to 4.18)	3.3 (2.66 to 4.11)	4.33 (3.5 to 5.36)
*H*_*post*_	0.08 (0.07 to 0.1)	0.42 (0.32 to 0.52)	0.4 (0.3 to 0.5)	0.38 (0.29 to 0.48)	0.39 (0.3 to 0.5)	0.13 (0.11 to 0.16)
Differences in variability						
between patients	No	No	No	No	No	Yes
*d* *f*_*pre*_	—	—	—	—	—	3.84 (3.06 to 4.82)
*d* *f*_*post*_	—	—	—	—	—	4.28 (3.4 to 5.38)
*ρ*_*Moran*_	—	—	—	—	—	0.37 (0.19 to 0.52)
*n*_*pars*_	13	15	19	23	27	26
*ℓ*	−31954.8	−31724.6	−31708.9	−31664.5	−31656.5	−31299.7^*a*^
*BIC*	64032.85	63591.41	63597.94	63547.06	63568.98	62845.9 ^*a*^

Parameter estimates are given with 95% confidence intervals in parentheses. ^*a*^Not comparable to other values in Table, as calculated using Laplace approximation. *A* *R* *T*_*t*_, time from seroconversion to treatment initiation; *BIC*, Bayesian information criterion; *ℓ*, log-likelihood; *NCS*, natural cubic spline; *n*_*pars*_, number of parameters estimated in model The interpretation of each model parameter is listed in Table 2

**Table 2:** Description of parameters for combined models of pre- and post-treatment data

*Model parameter*	*Description*					
*β*_0_	Pre-treatment mean intercept					
*β*_1_	Pre-treatment mean slope					
*U*_00_	Pre-treatment intercept subject-specific random effect variance					
*ρ*	Correlation between pre-treatment intercept and slope subject-specific random effects					
*U*_11_	Pre-treatment slope subject-specific random effect variance					
*σ*	Standard deviation of residual error term for each measurement, shared by pre- and post-treatment parts of model					
*κ*_*pre*_	Scale parameter for pre-treatment fBM process					
*H*_*pre*_	Hurst index for pre-treatment fBM process					
*ϕ*_1_ model	These parameters relate to the long-term maximum value of the response variable after treatment initiation					
*A* *t*1_1_,*A* *t*1_2_	Intercept and slope terms in relationship with $u_{i}^{+}$ for patients treated within 6 months of seroconversion					
*A* *t*2_1_,*A* *t*2_2_	Intercept and slope terms in relationship with $u_{i}^{+}$ for patients treated beyond 6 months but within 1 year of seroconversion					
*A*_1_,*A*_2_	Intercept and slope terms in relationship with $u_{i}^{+}$ for linear or NCS models ^*a*^					
*A*_3_,*A*_4_	Third and fourth coefficients for NCS models ^*a*^					
*ϕ*_2_ model	These parameters relate to the rate of recovery of the response variable after treatment initiation					
*B* *t*1_1_,*B* *t*1_2_	Intercept and slope terms in relationship with $u_{i}^{+}$ for patients treated within 6 months of seroconversion					
*B* *t*2_1_,*B* *t*2_2_	Intercept and slope terms in relationship with $u_{i}^{+}$ for patients treated beyond 6 months but within 1 year of seroconversion					
*B*_1_,*B*_2_	Intercept and slope terms in relationship with $u_{i}^{+}$ for linear or NCS models ^*a*^					
*B*_3_,*B*_4_	Third and fourth coefficients for NCS models ^*a*^					
*P*	Residual variance for long-term maximum (*ϕ*_1:*i*_) not explained by $u_{i}^{+}$					
*κ*_*post*_	Scale parameter for post-treatment fBM process					
*H*_*post*_	Hurst index for post-treatment fBM process					
*d* *f*_*pre*_	Degrees of freedom parameter for pre-treatment stochastic process					
*d* *f*_*post*_	Degrees of freedom parameter for post-treatment stochastic process					
*ρ*_*Moran*_	Correlation parameter for latent scaling variables of pre- and post-treatment stochastic processes					

^a^Only applicable to patients with treatment initiation more than 1year after seroconversion when separate terms are included for earlier groups. *fBM*, fractional Brownian motion; *NCS*, natural cubic spline Some of the parameters relate to the link functions between the ‘true’ value of the response variable at treatment initiation, $u_{i}^{+}$, and the post-treatment model

Fitting a model with separate linear relationships between $u_{i}^{+}$ and *ϕ*_1_ and *ϕ*_2_ according to timing of HAART subgroup (Model_4_) led to a reduction in BIC relative to the single-group natural cubic spines model. It was not possible to obtain a model fit for natural cubic spline functions defined separately for each subgroup (due to lack of convergence), but allowing linear functions in the early start subgroups in combination with natural cubic spline functions for the remaining patients led to a further improvement in model fit (Model_5_*vs* Model_4_, *Δ*2*ℓ* 16.0 for 4 parameters; *P*=0.003). However, Model_4_, with linear link functions for all subgroups, retained the lowest BIC value and so we have focused on interpretation of this model.

It is harder to make a direct comparison for Model_6_, which matches Model_4_ with the addition of jointly distributed latent scaling variables for the pre- and post-treatment fractional Brownian motion processes. Because of the need to integrate the log-likelihood function over multiple latent variables, parameter estimates for Model_6_ were obtained using the Laplace approximation, meaning that generalised likelihood ratio tests or comparisons of the BIC statistic are not appropriate. However, the low values obtained for the estimates of the pre- and post-treatment degrees of freedom parameters (which are effectively fixed at +*ve∞* for the other models considered) indicate that this model may better reflect the structure of the observed data. Convergence of parameter estimates was not achieved when the same extension was made to Model_5_.

Convergence of parameter estimates also failed when a subject-specific random effect was added to the speed of response to treatment function (*ϕ*_2_) for Model_4_, Model_5_ or Model_6_. We also attempted to extend each of these models to allow an independent linear effect of the patient-specific slope of pre-HAART decline (requiring an additional two latent variable per patient for their random intercept and slope terms), but convergence of parameter estimates was not achieved in each case. Using Model_4_, we checked the assumption that the pre- and post-HAART measurement error variance can be treated as constant, and no significant improvement in model fit was observed when separate parameters were fitted for the two periods (*Δ*2*ℓ* 0.6 for 1 parameter; *P*=0.44).

Plots of residuals derived from Model_6_ are provided in Additional file 1 (based on Fitzmaurice et al. [38] and Stirrup et al. [16]), and these do not indicate substantial problems with the fitted model. As a further check of the model structure developed, the fitted Model_6_ was used to simulate pre- and post-treatment CD4 counts for a cohort of 100 patients. The plot of these simulated data is visually consistent with the equivalent plot of 100 randomly selected patients from the real dataset. This comparison could be described as a posterior predictive check [39]. Additionally, a small simulation study was carried out to demonstrate that the use of a natural cubic spline basis for baseline CD4 count would be able to provide approximations to non-linear functions for the long-term maximum and speed of recovery following initiation of HAART, even if specification of the probability model as a whole is not completely correct; this is presented in Additional file 1. An R script and ADMB template files are also provided in Additional file 2 to simulate data based on the structure and point estimates of Model_6_, and to then refit Model_4_ and Model_6_ to these data.

### Model interpretation

**Fig. 4:**
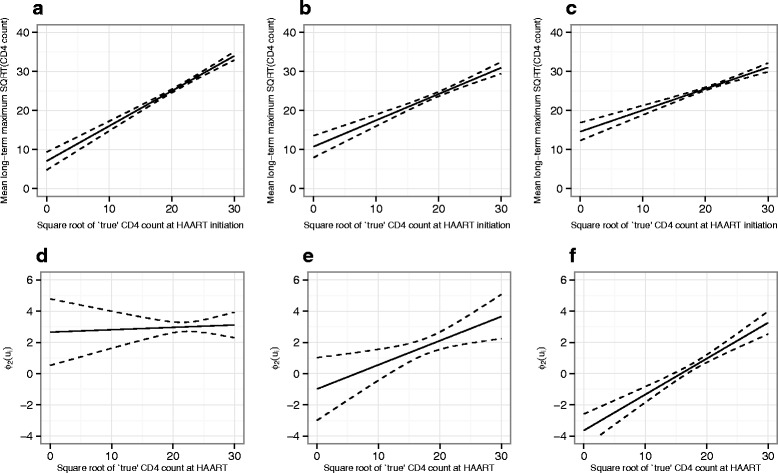
Plots of $\phi _{1}\left (u_{i}^{+} \right)$ (**a**–**c**, relating to long-term maximum) and $\phi _{2}\left (u_{i}^{+} \right)$ (**d**–**f**, relating to speed of response) for Model_4_. Graphs on the left of each row **a**, **d** show the fitted functions for patients initiating treatment within 6 months of seroconversion, those in the centre **b**, **e** show the functions for patients initiating treatment beyond 6 months but within 1 year and those on the right **c**, **f** show the functions for patients who started treatment beyond 1 year. Pointwise 95 % confidence intervals for the functions are shown as dashed lines

**Fig. 5:**
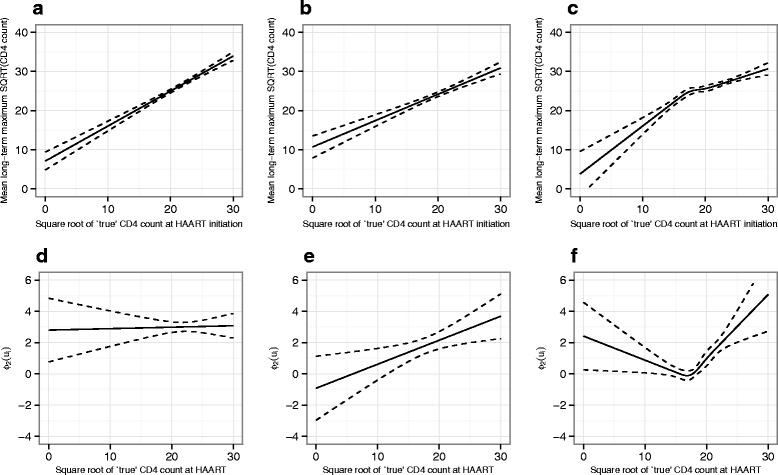
Plots of $\phi _{1}\left (u_{i}^{+} \right)$ (**a**–**c**, relating to long-term maximum) and $\phi _{2}\left (u_{i}^{+} \right)$ (**d**–**f**, relating to speed of response) for Model_5_. Graphs on the left of each row **a**, **d** show the fitted functions for patients initiating treatment within 6 months of seroconversion, those in the centre **b**, **e** show the functions for patients initiating treatment beyond 6 months but within 1 year and those on the right **c**, **f** show the functions for patients who started treatment beyond 1 year. Pointwise 95 % confidence intervals for the functions are shown as dashed lines

**Fig. 6:**
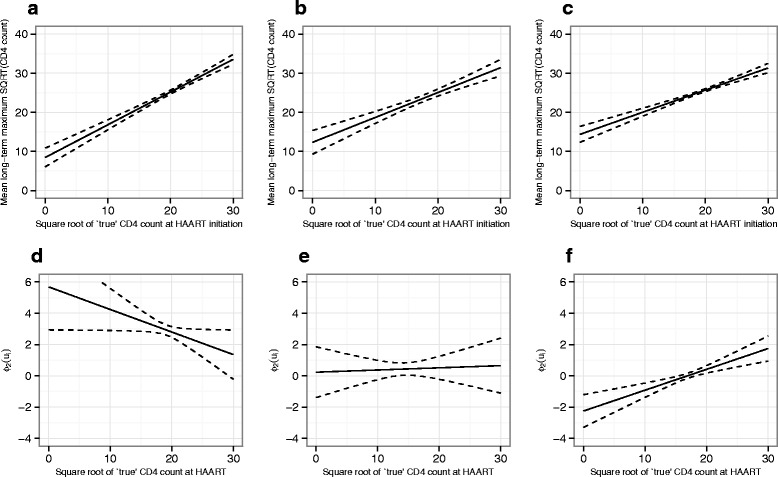
Plots of $\phi _{1}\left (u_{i}^{+} \right)$ (**a**–**c**, relating to long-term maximum) and $\phi _{2}\left (u_{i}^{+} \right)$ (**d**–**f**, relating to speed of response) for Model_6_. Graphs on the left of each row **a**, **d** show the fitted functions for patients initiating treatment within 6 months of seroconversion, those in the centre **b**, **e** show the functions for patients initiating treatment beyond 6 months but within 1 year and those on the right **c**, **f** show the functions for patients who started treatment beyond 1 year. Pointwise 95 % confidence intervals for the functions are shown as dashed lines

All models fitted (other than Model_1_ by definition) showed a positive association between baseline CD4 count at HAART and the long-term maximum; this finding was consistent across subgroups of patients defined by timing of treatment initiation with only relatively small differences in the fitted functions for each group in models 4–6 (Figs. 4, 5 and 6). When modelled as a linear function across all patients (i.e. Model_2_), the speed of response to treatment also showed a positive association with baseline CD4 count at HAART. However, when the link function was defined by HAART-timing subgroup, the speed of response to treatment was found to be substantially higher at moderate and lower baseline CD4 counts (below around 25 on the square-root scale) in those patients who started treatment within 6 months of seroconversion, with an intermediate difference observed for the subgroup who started treatment after 6 months but within 1 year. This overall pattern of findings was consistent across models 4–6, although the exact shape of the link functions showed some differences.

**Fig. 7:**
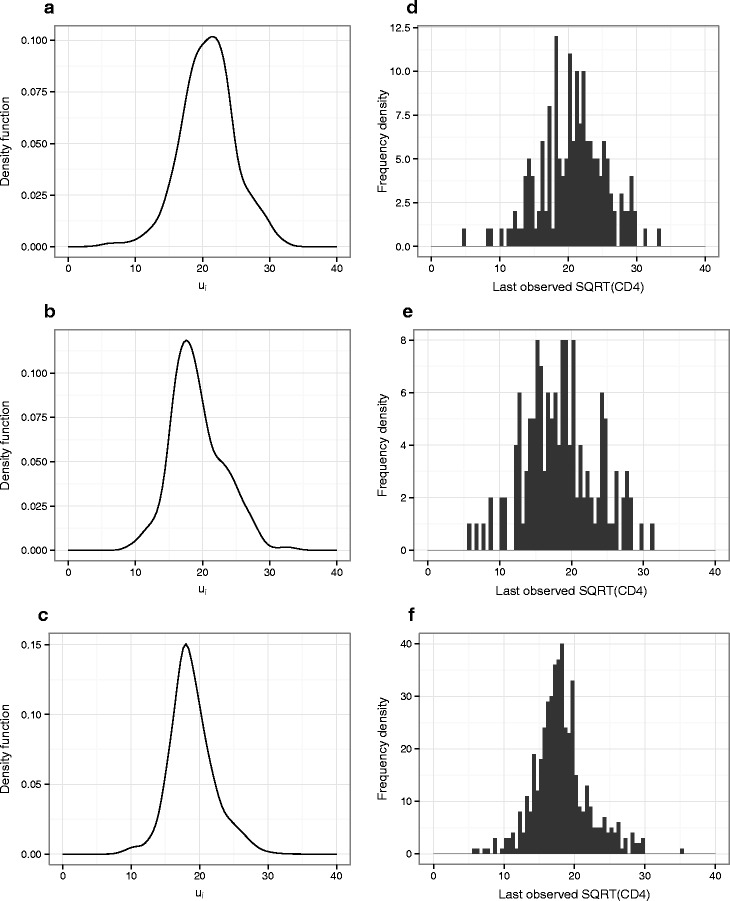
Kernel density plots (**a**–**c**) for the ‘true’ baseline square root CD4 counts and (**d**–**f**) histograms of the last observed square-root CD4 count before treatment. **a**–**c** Kernel density plots for the ‘true’ baseline square root CD4 counts for each individual (*u*_*i*_), approximating the posterior distribution of each as normal (with subject-specific standard deviation as estimated during model fitting), and **d**–**f** histograms of the last observed square-root CD4 count before treatment for those individuals in whom this was recorded within 6 months of treatment initiation (*n*=170, *n*=141 and *n*=486, respectively). Graphs in the top row **a**, **d** relate to patients initiating treatment within 6 months of seroconversion, those in the centre row **b**, **e** relate to patients initiating treatment beyond 6 months but within 1 year and those on the lower row **c**, **f** are for patients who started treatment beyond 1 year

As the full vector of pre- and post-treatment data and *u*_*i*_ for each individual do not jointly follow a multivariate normal distribution, it is not possible to derive a closed form for the posterior predictive distribution of the *u*_*i*_ conditioned on the observed data in the way that would be done for the realizations of the random effects in a linear mixed model. However, the values of *u*_*i*_ for each individual that maximise *f*(***y***_*pre*:*i*_,***y***_*post*:*i*_,*u*_*i*_), $\hat {u}_{i}$, conditional on the current values of the model parameters, are calculated at each iteration of the adaptive Gauss–Hermite quadrature algorithm. The values of $\hat {u}_{i}$ corresponding to the final parameter estimates for each model are returned by ADMB, and these correspond to the posterior mode of $f_{u|\boldsymbol {Y}_{pre}=\boldsymbol {y}_{pre}, \boldsymbol {Y}_{post}=\boldsymbol {y}_{post}} \left (u \right)$ for each individual. Kernal density plots for the *u*_*i*_ values for each subgroup in Model_4_ are presented in Fig. 7, approximating the distribution for $f_{u|\boldsymbol {Y}_{pre}=\boldsymbol {y}_{pre}, \boldsymbol {Y}_{post}=\boldsymbol {y}_{post}} \left (u \right)$ as normal and making use of subject-specific standard deviation estimates also resulting from the adaptive Gauss–Hermite quadrature algorithm. Equivalent plots for Model_5_ and Model_6_ did not show substantial differences. Histograms of the last observed square-root CD4 count before treatment for those individual in whom this was recorded within 6 months of treatment initiation are also presented in Fig. 7 for comparison, showing a similar shaped distribution in each subgroup. As expected given the results of previous simulations regarding treatment initiation based on observed CD4 cell counts [14], for more than half of patients (63 %) the mode of the posterior predictive distribution ($\hat {u}_{i}$) was greater than the last observed CD4 count (where available within 6 months); the median difference for $CD4_{last\_obs} - \hat {u}_{i}$ was −18 cells/ *μL* when transformed back to the original measurement scale.

**Fig. 8:**
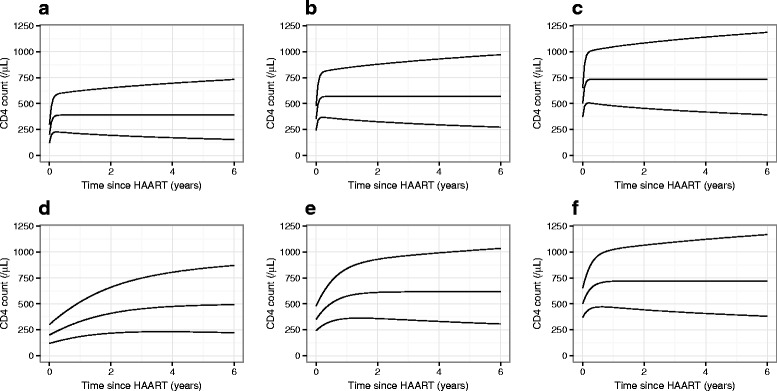
Predictions for hypothetical patients made from fitted model. Plots of the 90 % range of CD4 counts predicted by Model_4_ for a population of patients initiating highly active antiretroviral therapy (HAART) either within 6 months (**a**–**c**) or more than 1 year (**d**–**f**) from seroconversion, with ‘true’ CD4 counts at treatment initiation of: **a**, **d** 200, **b**, **e** 350 and **c**, **f** 500 cells/ *μ*L. The predicted ranges include measurement error (alongside the stochastic process component and variance in the subject-specific long-term maximum), explaining the variance present at time zero. The ranges shown have been back-transformed from the model predictions generated on the square-root scale

Predicted ranges for CD4 cell counts based on Model_4_ are shown in Fig. 8 for patients with a ‘true’ CD4 counts at initiation of HAART of 200, 350 and 500 cells/ *μL*. These charts further illustrate the model predictions that, in general, patients with a higher CD4 cell count at treatment initiation will go on to show a higher long-term maximum and will attain higher values more quickly after the start of treatment, but that response to treatment is rapid if it is initiated within 6 months of seroconversion regardless of baseline CD4. These charts also illustrate that the model predicts considerable variability in response to treatment between patients at any given baseline CD4 value. However, in the models presented we have not included variables such as patient age, gender and mode of infection that may also be predictive of response to treatment, and so it is possible that more fully developed models would include less unexplained variance in the long-term response to treatment. The inclusion of such potential confounding variables may also affect estimates of the influence of baseline value of CD4 at treatment initiation on each patient’s response to treatment. Equivalent plots for Model_5_ and Model_6_ showed similar overall patterns of predictions.

**Fig. 9:**
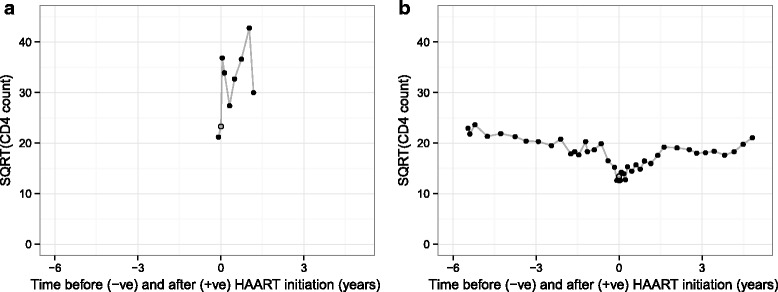
Plots of CD4 counts (∙) observed in the two patients with the most (**a**) and least (**b**) erratic response to highly active antiretroviral therapy (HAART). Variability of response was assessed as indicated by the modes of the posterior predictive distributions of the post-treatment latent scaling variables ($\widehat {w_{2:i}}$) obtained from Model_6_. The mode of the posterior predictive distribution for the ‘true’ baseline value ($\widehat {u_{i}}$) is also shown in each case (∘)

For Model_6_, estimates of the pre- and post-treatment degrees of freedom parameters (3.84 (95 % CI, 3.06–4.82) and 4.28 (3.4–5.38), respectively) indicate that there are considerable between-patient differences in the variability of observations over time. It is interesting to note that the correlation parameter between the pre- and post-treatment latent scaling variables was positive, but only of moderate magnitude ($\hat {\rho }_{Moran}$0.37 (0.19–0.52)), i.e. the degree of variability over time before and after treatment for each patient shows a moderate positive correlation. It is also of interest that the estimated H-index for the post-treatment fractional Brownian motion process in this model was much lower than that for the equivalent model without the latent scaling variables (0.13 (0.11–0.16) *vs* 0.38 (0.29–0.48)), indicating that although some patients show high variability in CD4 observations over time, successive increments of the stochastic process are strongly negatively correlated and there is an associated reversion of the process towards the underlying mean in each patient. It is possible to use the modes of the posterior predictive distributions of the latent scaling variables for each patient to identify those individuals with particularly smooth or erratic patterns of CD4 counts over time; observations for the two patients with the most extreme values obtained for the post-treatment latent scaling variable are plotted in Fig. 9.

## Discussion

The statistical methodology developed in this article provides a novel framework for the combined analysis of pre- and post-treatment longitudinal biomarker data. The approach proposed has the advantage of making use of all available data, does not require an a priori assumption regarding the distribution of baseline values at treatment across the studied population as a whole and allows a flexible choice of functions to link the pre- and post-treatment trajectories of the biomarker under investigation for each patient. When applied to CD4 data from the UK Register of Seroconverters cohort, the resulting fitted models provide evidence of a positive association between baseline CD4 count at initiation of HAART and the long-term maximum achieved by each patient, which is consistent with previous published literature on this topic [9–11]. In addition the fitted models suggest that initiation of HAART closer to the date of HIV seroconversion is associated with a more rapid response to treatment, regardless of the baseline CD4 value. This finding warrants further investigation in larger datasets, with inclusion of additional factors that are thought to be associated with response to treatment into the modelling framework; this extension would be straightforward using the methodology developed.

The standard non-linear mixed effects model approach in this situation, ignoring observations before the start of treatment, would require rigid assumptions regarding the distribution of the biomarker variable at treatment initiation and its relationship to subsequent post-treatment observations, i.e. typically that baseline values and the long-term maximum value for each patient follow a bivariate normal distribution. The modelling strategy that we have developed allows greater flexibility in the link between baseline and post-treatment maximum values of the biomarker, and does not restrict the shape of the overall marginal distribution of baseline values in the studied population. Alternatively, the standard use of baseline observations as a predictive variable would also discard any information from measurements obtained prior to this point in time and would require a separate imputation model for missing values of the baseline measurement, which would not be straightforward to define for observational data with highly irregular number and timing of measurements for each patient. Furthermore, it is not obvious how the primary model for multiple post-treatment observations should be structured in this context, as it would be overly restrictive to assume a constant fixed effect coefficient for the baseline observation for all time points after the initiation of treatment.

The proposed model for the analysis of pre- and post-treatment CD4 data has been structured so that the estimated parameters of the different components of the model each have a clear practical interpretation, i.e. it is of direct interest to clinicians to know how baseline CD4 and time from seroconversion at initiation of HAART are associated with the speed and maximal level of treatment response that can be expected. If further patient variables were added to the functions that determine the characteristics of response to treatment then the modelled effects would be independent of the influence of the true baseline value of the biomarker, making interpretation of estimated coefficients relatively simple. If a mixed effects model is fitted to only baseline and post-treatment measurements, then assessment of the influence of a covariable on treatment response conditional on a baseline observation requires an additional stage of statistical adjustment [40].

The cost of using a combined model for pre- and post-treatment data is that we are required to assume that the proposed model structure provides an adequate description of the data under analysis. The requirement for strong assumptions regarding the correctness of model structure has been used as an argument against the use of integrated models for baseline and treatment response data [3]. In the present study, the motivations for the inclusion of pre- and post-treatment stochastic process components in the models and for the use of natural cubic spline functions to link baseline CD4 and characteristics of the treatment response trajectory were to maximise model flexibility and therefore provide an optimal fit to the data. However, we plan to investigate further extensions of the model structure using larger datasets, which would be able to support a greater number of parameters in model-fitting. As such, the scientific results from the present study can only be taken as preliminary findings.

An advantage of the extension of the non-linear mixed effects modelling approach as developed in this paper is that the nature of the variability in biomarker observations over time within each patient can be investigated, whereas this is often lost when using approaches that only consider population mean values or the marginal distribution of observations across the population at each point in time. A focus on realistic modelling of the patterns of variation in the data is also required in order to provide valid inference under the ‘missing at random’ assumption for missing data and when the timing of observations is dependent on previous outcomes [35]. A limitation of the present analysis is that we have not considered the possibility of censoring being related to underlying latent variable terms rather than just the observed CD4 counts. Such joint modelling of longitudinal and event time data [41, 42] would provide useful information regarding the patterns of drop-out from the cohort, but would add further to the computational complexity of estimation.

The fitted models in the present analysis show that there is considerable unexplained variance in the long-term asymptotic maximal response to treatment for each patient, even after accounting for baseline CD4 and time from seroconversion to initiation of HAART, although this might be reduced by the inclusion of additional patient and drug regimen variables into the model. There is also considerable erratic post-treatment variability over time, represented by the fractional Brownian motion process as previously introduced for the analysis of pre-treatment CD4 data [16]. The parameter estimates for the model in which the stochastic process components were generalised to follow marginal multivariate t-distributions indicate substantial between-patient differences in their variability over time, with a moderate positive association between the degree of pre- and post-treatment variability within each patient, which are novel findings in this context. The fact that the models fitted follow a structure that can accommodate any combination of number and timing of observations in each patient means that they can be readily used for simulation studies of patient cohorts.

## Conclusions

We have developed a framework for the combined analysis of pre- and post-treatment longitudinal biomarker data and have successfully applied the novel methodology to CD4 data from a cohort of HIV-positive patients with well estimated date of seroconversion. The methodology developed could also be applied to other medical settings in which an intervention is triggered following monitoring of a biomarker of interest, and in which the response to treatment may be conditional on the state of the patient (as indicated by the value of the biomarker) at the time of treatment initiation. Seroconverter cohorts have a special status in HIV research, and in other disease settings the ‘zero time’ for pre-treatment observations might be time of diagnosis or another clinically significant event. The framework proposed could be applied with different choice of pre- and post-treatment model components, but those demonstrated may be a natural choice in many settings.

## Abbreviations

ADMB, AD model builder; AIDS, acquired immune deficiency syndrome; BIC, Bayesian information criterion; HAART, highly active antiretroviral therapy; HIV, human immunodeficiency virus; IQR, interquartile range; MVN, multivariate normal; RCT, randomised controlled trial; UCL, University College London; UK, United Kingdom

## Additional files

Additional file 1 Appendices containing (1) details of marginal distribution for post-treatment model and coding for positive-only latent variable, (2) residual plots for Model_6_ and (3) results of a simulation study demonstrating approximation of non-linear link functions using natural cubic splines. (PDF 1290 kb)

Additional file 2 Tar file containing an R script and ADMB template files to simulate data based on the structure and point estimates of Model_6_, as described in Results section, and to then refit Model_4_ and Model_6_ to these data. (TAR 205 kb)
