# Supplementary material for: Combined models for pre- and post-treatment longitudinal biomarker data: an application to CD4 counts in HIV-patients
Source: BMC Med Res Methodol. 2016 Sep 15;16:121. doi: 10.1186/s12874-016-0187-2 (PMC5025623; doi:10.1186/s12874-016-0187-2)
Supplement: Additional file 1 — Appendices containing (1) details of marginal distribution for post-treatment model and coding for positive-only latent variable, (2) residual plots for Model6 and (3) results of a simulation study demonstrating approximation of non-linear link functions using natural cubic splines. (PDF 1290 kb) [file 12874_2016_187_MOESM1_ESM.pdf]

1 Computational notes

Marginal distribution for post-treatment model

Although the models that we have defined for the post-treatment data are non-linear in their parameters, they are all linear in their random terms conditional on the value of u_i^+:

$$\begin{aligned}
\mathbf{y}_{post:i}|_{U_i^+=u_i^+} &= \mathbf{g}\left(\mathbf{t}_{post:i}, u_i^+, \tau_i\right) + \mathbf{W}_{post:i} + \mathbf{e}_{post:i} \\
&= \begin{pmatrix} \phi_{1:i} + \left(u_i^+ - \phi_{1:i}\right)\exp\left(-\exp\left(\phi_{2:i}\right) t_{post:i1}\right) \\ \vdots \\ \phi_{1:i} + \left(u_i^+ - \phi_{1:i}\right)\exp\left(-\exp\left(\phi_{2:i}\right) t_{post:in_{post:i}}\right) \end{pmatrix} + \mathbf{W}_{post:i} + \mathbf{e}_{post:i} \\
&= \begin{pmatrix} \phi_1\left(u_i^+\right) + \tau_i + \left(u_i^+ - \left(\phi_1\left(u_i^+\right) + \tau_i\right)\right)\exp\left(-\exp\left(\phi_2\left(u_i^+\right)\right) t_{post:i1}\right) \\ \vdots \\ \phi_1\left(u_i^+\right) + \tau_i + \left(u_i^+ - \left(\phi_1\left(u_i^+\right) + \tau_i\right)\right)\exp\left(-\exp\left(\phi_2\left(u_i^+\right)\right) t_{post:in_{post:i}}\right) \end{pmatrix} + \mathbf{W}_{post:i} + \mathbf{e}_{post:i}
\end{aligned}$$

$$\begin{aligned}
\tau_i &\sim N(0, P) \\
\mathbf{W}_{post:i} &\sim MVN(\mathbf{0}, \mathbf{\Sigma}_{post:i}) \\
\mathbf{e}_{post:i} &\sim MVN(\mathbf{0}, \sigma^2 \mathbf{I}_{n_{post:i}}).
\end{aligned}$$

As such, for the models defined, the post-treatment data follows a marginal multivariate normal distribution conditional on the value of u_i^+, with mean vector given by:

$$\mathrm{E}\left[\mathbf{y}_{post:i}\right]_{|U_i^+=u_i^+} = \begin{pmatrix} \phi_1\left(u_i^+\right) + \left(u_i^+ - \phi_1\left(u_i^+\right)\right)\exp\left(-\exp\left(\phi_2\left(u_i^+\right)\right) t_{post:i1}\right) \\ \vdots \\ \phi_1\left(u_i^+\right) + \left(u_i^+ - \phi_1\left(u_i^+\right)\right)\exp\left(-\exp\left(\phi_2\left(u_i^+\right)\right) t_{post:in_{post:i}}\right) \end{pmatrix},$$

and covariance matrix given by:

$$\mathrm{Var}\left[\mathbf{y}_{post:i}\right]_{|U_i^+=u_i^+} = \mathbf{Q}_i + \mathbf{\Sigma}_{post:i} + \sigma^2 \mathbf{I}_{n_{post:i}},$$

where the jk^{th} element of $\mathbf{Q}_i$, $q_{i_{jk}}$, is given by:

$$\begin{aligned}
q_{i_{jk}} &= \mathrm{Cov}\left[\left(1 - \exp\left(-\exp\left(\phi_2\left(u_i^+\right)\right) t_{post:ij}\right)\right)\tau_i, \left(1 - \exp\left(-\exp\left(\phi_2\left(u_i^+\right)\right) t_{post:ik}\right)\right)\tau_i\right] \\
&= \left(1 - \exp\left(-\exp\left(\phi_2\left(u_i^+\right)\right) t_{post:ij}\right)\right) \times \left(1 - \exp\left(-\exp\left(\phi_2\left(u_i^+\right)\right) t_{post:ik}\right)\right) \times P.
\end{aligned}$$

Coding for positive-only latent variable

The ADMB software is designed to find maximum likelihood estimates for a function that is differentiable in terms of all of its parameters and latent variables. Because of this conditional statements, such as `if/then` statements and `max(a,b)`, involving the values of parameters or latent variables are not supported. Instead, a very steep logistic function is used to obtain u_i^+ from u_i:

```
double high_c = 1000000;
dvariable u_pos = u/(1 + mfexp(-high_c*u));
```

The `double` defined here is a standard C++ double-precision floating point constant, whilst the `dvariable` is a class of object specific to ADMB that is designed to store the necessary information regrading gradient structure for optimisation.

It is also worth noting that we use the function `mfexp(value)` in place of `exp(value)` throughout, as the former is an adjusted version of the standard function that guards against numerical under- or overflow for large absolute values of the function argument ($|value| > 60$). This can particularly be a problem when dealing with nested exponential functions.

2 Evaluation of residuals

We present here plots of residuals obtained from the fit of Model_6 to the UK Register of Seroconverters dataset. The approach taken is similar that used previously by Stirrup *et al.*[1] in assessing models for pre-treatment CD4 cell counts alone, which in turn was developed from suggestions made by Fitzmaurice, Laird and Ware[2]. Firstly we note that, for pre-treatment CD4 cell counts, the distribution for the full set of observations for each patient is multivariate normal conditional on the value of the latent scaling variable associated with the pre-treatment fractional Brownian motion process:

$$\begin{aligned}
\mathbf{y}_{pre:i} &= \mathbf{X}_i\boldsymbol{\beta} + \mathbf{Z}_i\mathbf{b}_i + \mathbf{W}_{pre:i} + \mathbf{e}_{pre:i} \\
\mathbf{b}_i &\sim MVN(\mathbf{0}, \boldsymbol{\Psi}) \\
\mathbf{W}_{pre:i}|_{W_{1:i}=w_{1:i}} &\sim MVN(\mathbf{0}, \frac{1}{w_{1:i}}\boldsymbol{\Sigma}_{pre:i}) \\
\mathbf{e}_{pre:i} &\sim MVN(\mathbf{0}, \sigma^2\mathbf{I}_{n_{pre:i}}).
\end{aligned}$$

We can therefore obtain an estimate of the pre-treatment marginal covariance matrix specific to each patient based on the posterior predictive mode of their latent scaling variable, $\hat{w}_{1:i}$:

$$\hat{\mathbf{V}}_{pre:i} = \mathbf{Z}_i\hat{\boldsymbol{\Psi}}\mathbf{Z}_i^{\mathrm{T}} + \frac{1}{\hat{w}_{1:i}}\hat{\boldsymbol{\Sigma}}_{pre:i} + \hat{\sigma}^2\mathbf{I}_{n_{pre:i}}.$$

If the model parameters and the value of the scaling variable were known, then the distribution of the transformed marginal residuals using the inverse of the Cholesky decomposition of the covariance matrix for each individual,$\mathbf{V}_{pre:i}$, would be normally and independently distributed with mean $\mathbf{0}$ and variance $\mathbf{1}$:

$$\begin{aligned}
\mathbf{V}_{pre:i} &= \mathbf{L}_i\mathbf{L}_i^{\mathrm{T}} \\
\mathbf{L}_i^{-1}\left(\mathbf{y}_i - \mathbf{X}_i\boldsymbol{\beta}\right) &\sim MVN\left(\mathbf{0}, \mathbf{I}_{n_i}\right).
\end{aligned}$$

Plots of the Cholesky-transformed residuals of pre-treatment observations for Model_6, with the covariance matrix estimated for each patient based on the posterior predictive mode of their latent scaling variable, $\hat{w}_{1:i}$, are presented in Figures 1 and 2.

For post-treatment observations, the distribution for the full set of observations for each patient is multivariate normal conditional on the value of both the true baseline CD4 value and the latent scaling variable associated with the post-treatment fractional Brownian motion process:

$$\begin{aligned}
\mathbf{y}_{post:i}|_{U_i^+=u_i^+} &= \mathbf{g}\left(\mathbf{t}_{post:i}, u_i^+, \tau_i\right) + \mathbf{W}_{post:i} + \mathbf{e}_{post:i} \\
\tau_i &\sim N(0, P) \\
\mathbf{W}_{post:i}|_{W_{2:i}=w_{2:i}} &\sim MVN(\mathbf{0}, \frac{1}{w_{2:i}}\boldsymbol{\Sigma}_{post:i}) \\
\mathbf{e}_{post:i} &\sim MVN(\mathbf{0}, \sigma^2\mathbf{I}_{n_{post:i}}).
\end{aligned}$$

As noted in Appendix 1 this forms a multivariate normal distribution conditional on u_i^+ and $w_{2:i}$, given that $\mathbf{g}\left(\mathbf{t}_{post:i}, u_i^+, \tau_i\right)$ is linear in τ_i. As such, a covariance matrix can be

constructed for the post-treatment observations of each patient based on the posterior predictive modes of u_i^+ and $w_{2:i}$, and Cholesky-transformed residuals can be calculated as for the pre-treatment data. Such plots are presented for Model_6 Figures 3 and 4.

The residual plots shown in Figures 1–4 do not indicate any substantial model misspecification. However, following Stirrup *et al.*[1] we also consider plots summarising Cholesky-residuals conditional on multiple samples from the joint posterior predictive distribution of the latent variables, using the approximate multivariate normal distribution as returned by ADMB. For the latent scaling variables relating to the pre- and post-treatment stochastic process components of the model, sampling was based on the bivariate normal a and b variables as used for the Laplace approximation of the integral, with transformation to the necessary gamma variates as described in the paper. Plots equivalent to Figures 1–4, but based on 1000 sets of samples, are shown in Figure 5. These plots are similar to those based on the posterior predictive modes of the latent variables, and so provide further evidence for adequacy of the model fit.

As a further check of the model structure developed, the fitted Model_6 was used to simulate pre- and post-treatment CD4 counts of a cohort of 100 patients. As we have not developed a probabilistic model for the timing of initiation of treatment, and in order to generate a range of different conditions, these patients were randomised to initiate treatment either: (1) immediately at the time of seroconversion, (2) 1 year after seroconversion or at the first observation below (3) 500, (4) 350 or (5) 200. Data were generated on the square-root(CD4) scale, and cut-off points for initiation of treatment were accordingly transformed to this scale. Each patient, up until the point of treatment initiation, was scheduled to be observed at 4-month intervals from seroconversion; treatment was initiated at 8 years if the threshold for a specific patient had not been triggered before this point. Following treatment initiation, observations were simulated after 1, 2, 3 and 4 months, and at 4-month intervals thereafter up until a maximum of 5 years. An R-script to generate such a cohort is provided as supporting information to this paper, along with ADMB template files to refit Model_4 and Model_6 to the simulated dataset. A plot of CD4 counts from the simulated cohort is provided in Figure 6. This plot is visually consistent with the equivalent plot of 100 randomly selected patients from the real dataset, although in the artificial dataset no allowance has been made for irregular timing of observations or of loss-to follow-up or administrative censoring of patients. This comparison could be described as a posterior predictive check[3].

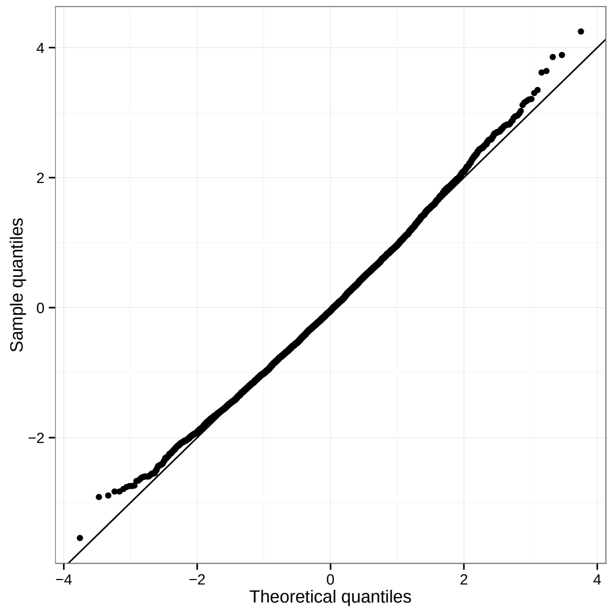

Figure 1 Quantile–quantile plot of Cholesky-transformed residuals of pre-treatment observations for Model_6, calculated using the posterior predictive mode of the latent scaling variable $\hat{w}_{1:i}$ for each patient.

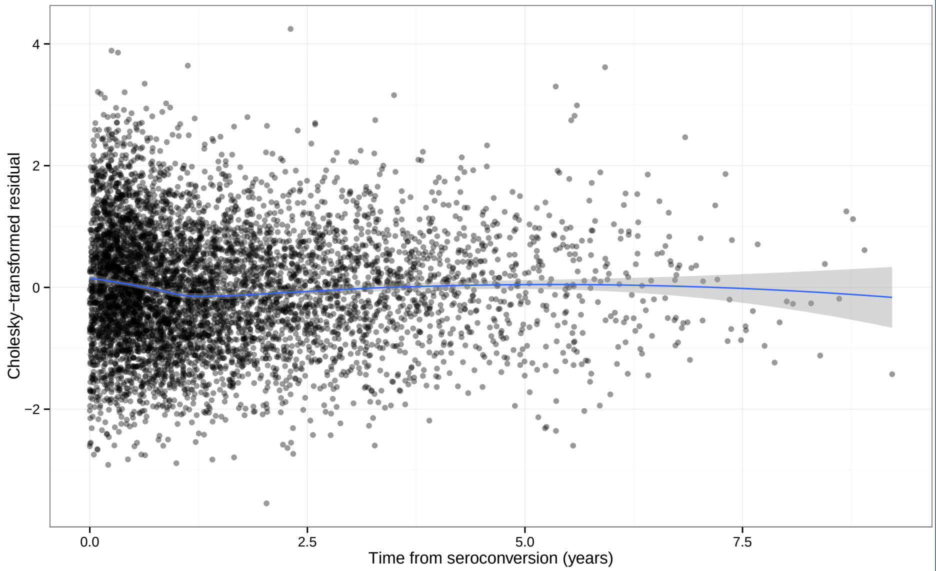

Figure 2 Plot of Cholesky-transformed residuals of pre-treatment observations for Model_6 against time from seroconversion, calculated using the posterior predictive mode of the latent scaling variable $\hat{w}_{1:i}$ for each patient. A LOESS regression line with 95 % confidence interval has been added.

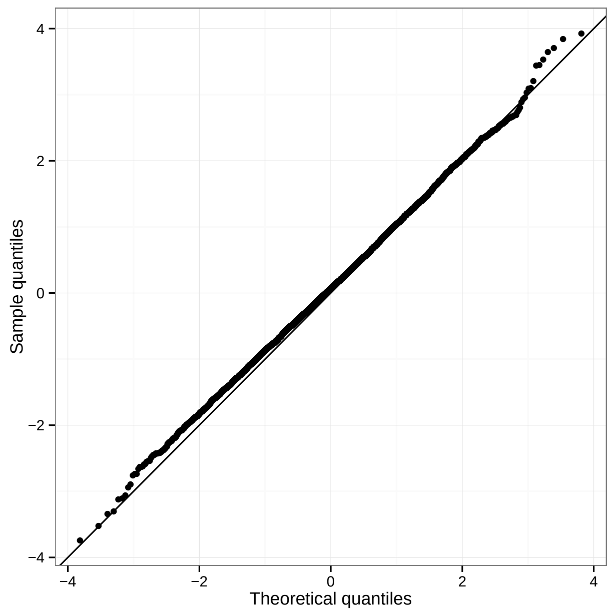

Figure 3 Quantile–quantile plot of Cholesky-transformed residuals of post-treatment observations for Model$_6$, calculated using the posterior predictive mode of the baseline CD4 value $\hat{u}_i$ and the latent scaling variable $\hat{w}_{2:i}$ for each patient.

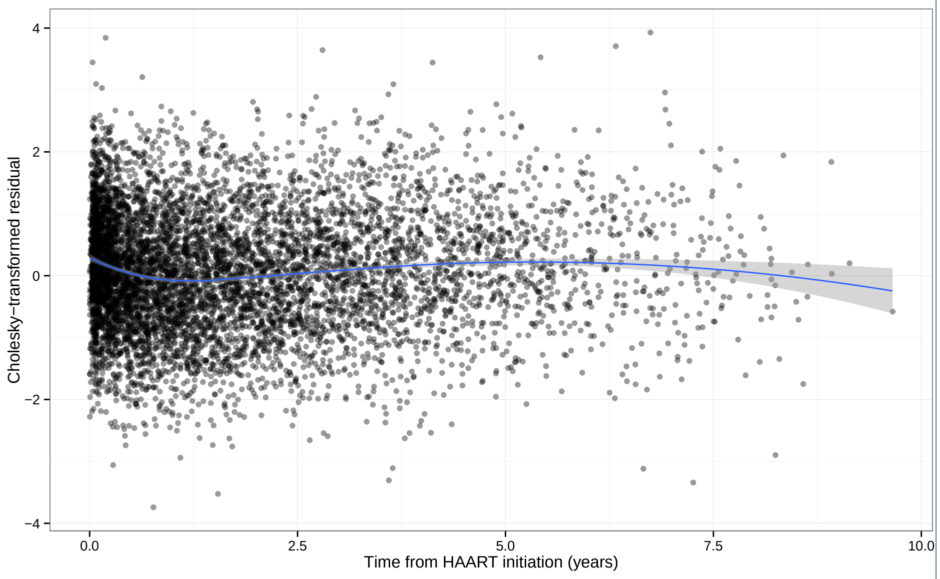

Figure 4 Plot of Cholesky-transformed residuals of post-treatment observations for Model$_6$ against time from initiation of HAART, calculated using the posterior predictive mode of the baseline CD4 value $\hat{u}_i$ and the latent scaling variable $\hat{w}_{2:i}$ for each patient. A LOESS regression line with 95 % confidence interval has been added.

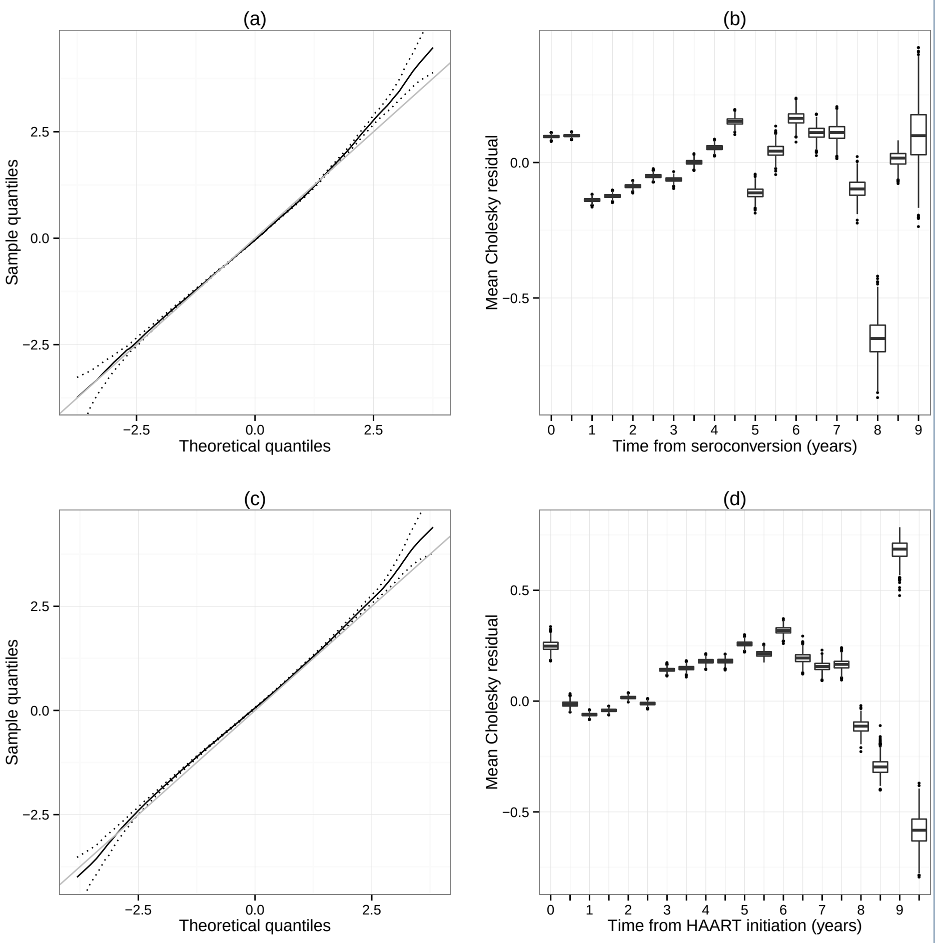

Figure 5 Plots of Cholesky-transformed residuals of pre- (a,b) and post- (c,d) treatment observations for Model_6, calculated based on 1000 draws from the approximate posterior predictive distribution of the latent variables for each patient. The quantile–quantile plots (a,c) show the 2.5th, 50th and 97.5th percentiles of the sample quantiles for each theoretical quantile corresponding to the total number of observations. (b) and (d) show boxplots of the mean residual across repeated samples, with observations grouped according to the nearest multiple of 6 months.

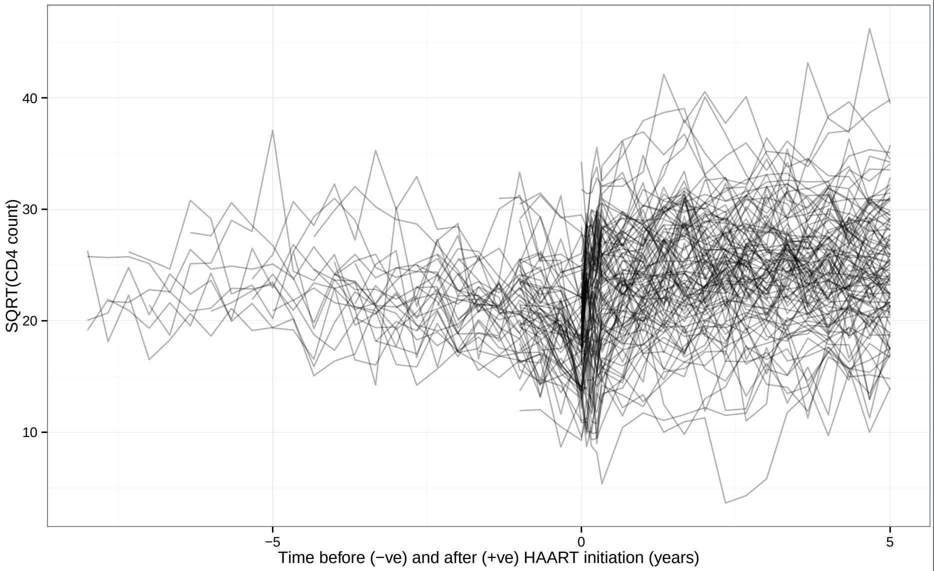

Figure 6 Plot of CD4 counts relative to the initiation of HAART for a simulated cohort of 100 patients based on Model_6.

3 Model fitting to simulated data

In order to check that the use of natural cubic splines would be able to recover non-linear functions for $\phi_1\left(u_i^+\right)$ and $\phi_2\left(u_i^+\right)$, even if the probability model as a whole were not correctly specified, we simulated cohorts of patients based on a modified version of Model_6. The point estimates of parameters were used as obtained from the UK Register of Seroconverters dataset, but to simplify the analysis the recovery of CD4 counts after initiation of treatment was assumed to depend only on the 'true' CD4 value at baseline and *not* on the time elapsed from seroconversion to initiation. Furthermore, $\phi_1\left(u_i^+\right)$ and $\phi_2\left(u_i^+\right)$ were modified to follow non-linear sigmoidal functions:

$$\phi_1\left(u_i^+\right) = 15 + \frac{15}{1+\exp(-0.5 * (u_i^+ - 15))}$$
$$\phi_2\left(u_i^+\right) = \frac{2}{1+\exp(-0.5 * (u_i^+ - 20))}.$$

Twelve cohorts of 250 patients were generated, using the observation and treatment initiation schedule as described in Appendix 2, and Model_3 was fitted to each cohort — i.e. with a natural cubic spline function to approximate $\phi_1\left(u_i^+\right)$ and $\phi_2\left(u_i^+\right)$, without any dependence on the time from seroconversion to treatment initiation. The sample size was chosen for convenience, as the maximum number of separate processes that could be initiated from R using the cluster system available. Convergence of maximum likelihood estimates of the model parameters was achieved for 10/12 of these simulated cohorts. The fitted functions for $\phi_1\left(u_i^+\right)$ and $\phi_2\left(u_i^+\right)$ in each case are shown in Figures 7 and 8, respectively. A histogram of the 'true' CD4 values at treatment initiation for each patient in the first cohort is shown in Figure 9.

The plots of the fitted functions for $\phi_1\left(u_i^+\right)$ and $\phi_2\left(u_i^+\right)$ indicate that natural cubic splines can be used to approximate non-linear relationships between latent variables, even if the probability model as a whole is not completely correctly specified. However, the natural cubic splines are constrained to a linear function beyond the upper and lower boundary knots, and this clearly affects the ability of the approach to model response to treatment in patients with very high or very low baseline CD4 at treatment initiation. Adding more knots to the natural cubic spline basis would allow more flexibility in the fitted function, but at the cost of reduced computational stability. Hence these plots indicate that caution is required when interpreting predictions or attempting to draw inferences regarding patients with unusually high or low CD4 values at treatment initiation, and reinforce the general principle that fitted relationships should not be extrapolated beyond the range of values observed in the dataset under analysis.

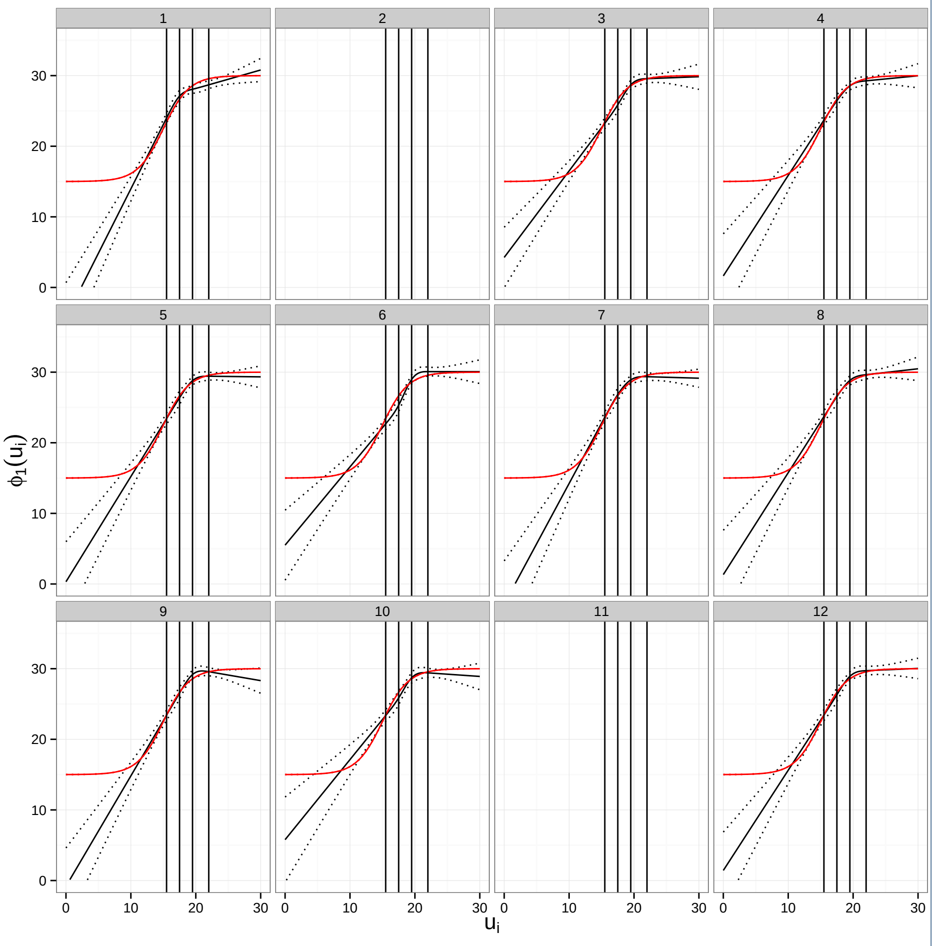

Figure 7 Plots of estimates of $\phi_1\left(u_i^+\right)$ (black curved line, with dotted 95 % CI) obtained by fitting Model_3 to 12 simulated cohorts of 250 patients. The function specified as used to generate the data is shown in red. The vertical black lines show the positions of knots for restricted natural cubic spline basis.

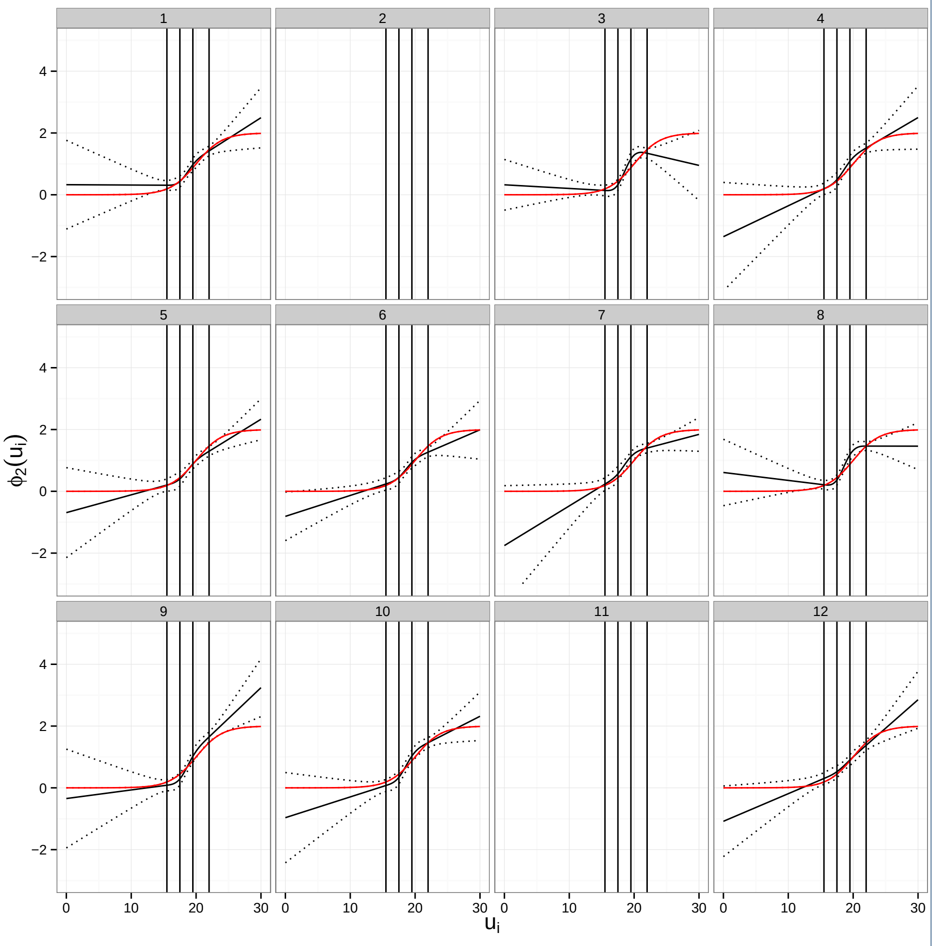

Figure 8 Plots of estimates of $\phi_2\left(u_i^+\right)$ (black curved line, with dotted 95 % CI) obtained by fitting Model$_3$ to 12 simulated cohorts of 250 patients. The function specified as used to generate the data is shown in red. The vertical black lines show the positions of knots for restricted natural cubic spline basis.

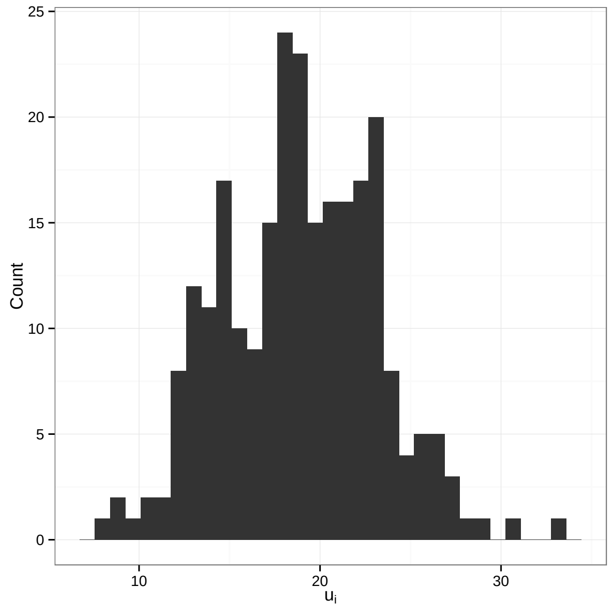

Figure 9 Histogram of 'true' CD4 values at treatment initiation for each patient in the first cohort of the simulation study.

References

1. Stirrup, O.T., Babiker, A.G., Carpenter, J.R., Copas, A.J.: Fractional Brownian motion and multivariate-t models for longitudinal biomedical data, with application to CD4 counts in HIV-patients. Statistics in Medicine **35**, 1514–1532 (2016)
2. Fitzmaurice, G., Laird, N., Ware, J.: Residual analyses and diagnostics. In: Applied Longitudinal Analysis. Wiley, Hoboken, NJ (2004)
3. Gelman, A.: Exploratory data analysis for complex models. Journal of Computational and Graphical Statistics **13**, 755–779 (2004)
